# Linking land use and precipitation changes to water quality changes in Lake Victoria using earth observation data

**DOI:** 10.1007/s10661-024-13261-2

**Published:** 2024-10-25

**Authors:** Maria Theresa Nakkazi, Albert Nkwasa, Analy Baltodano Martínez, Ann van Griensven

**Affiliations:** 1 Department of Water and Climate, Vrije Universiteit Brussel (VUB), 1050 Brussels, Belgium; 2 Water Security Research Group, Biodiversity and Natural Resources Program, International Institute for Applied Systems Analysis (IIASA), Schlossplatz 1, A-2361 Laxenburg, Austria; 3 Water Science & Engineering Department, IHE Delft Institute for Water Education, 2611 AX Delft, The Netherlands

**Keywords:** Climate change, Land use change, Remote sensing, Lake Victoria

## Abstract

Due to the continued increase in land use changes and changing climatic patterns in the Lake Victoria basin, understanding the impacts of these changes on the water quality of Lake Victoria is imperative for safeguarding the integrity of the freshwater ecosystem. Thus, we analyzed spatial and temporal patterns of land cover, precipitation, and water quality changes in the Lake Victoria basin between 2000 and 2022 using global satellite products. Focusing on chlorophyll-a (Chl-a) and turbidity (TUR) in Lake Victoria, we used statistical metrics (correlation coefficient, trend analysis, change budget, and intensity analysis) to understand the relationship between land use and precipitation changes in the basin with changes in Chl-a and TUR at two major pollution hotspots on the lake, i.e., Winam Gulf and Inner Murchison Bay (IMB). Results show that the Chl-a and TUR concentrations in the Winam gulf increase with increases in precipitation. Through increases in precipitation, the erosion risks are increased and transport of nutrients from land to the lake system, promoting algal growth and turbidity. In the IMB, Chl-a and TUR concentrations decrease with an increase in precipitation, possibly due to dilution, but peak during moderate rainfall. Interestingly, changes in land use and land cover (LULC) at 5-year intervals showed no substantial correlation with water quality changes at selected hotspots even though a broader LULC change analysis over the past two decades indicated a notable 300% increase in built-up areas across the Lake Victoria basin. These findings underscore the dominant influence of precipitation changes over LULC changes on the water quality of Lake Victoria for the selected hotspot areas.

**Supplementary Information:**

The online version contains supplementary material available at 10.1007/s10661-024-13261-2.

## Introduction

The deterioration of water quality (WQ) in freshwater bodies is a pervasive and escalating global issue, detrimentally impacting ecosystems (Kundu et al., 2017; Peters & Meybeck, 2000). Contributory factors to this decline encompass the continual rise in population, urbanization, industrialization, and the influence of climate change (Bhateria & Jain, 2016; Me et al., 2018; Razman et al., 2023). Over the last 50 years, Lake Victoria, the largest tropical freshwater lake in the world, has faced threats from nutrient sources such as surface runoff, wastewater, agricultural waste, and atmospheric deposition, endangering both local communities and its biodiversity (Kayombo & Jorgensen, 2006). The lake’s ecosystem has experienced significant and alarming changes such as algal blooms, declining water transparency, water hyacinth, over-fishing, introduction of exotic fish species, and oxygen depletion (Achieng, 1990; Aloyce et al., 2001; Njiru et al., 2008). These changes can be attributed to high population densities that put a strain on the lake’s natural resources, resulting in land degradation (Wang et al., 2012). This has in turn impacted the hydrology of multiple rivers in the basin and, as a result, affected the lake’s dynamics (Olang & Fürst, 2011).

Changes in land use, which are largely controlled by human activities, have played a substantial role in the degradation of the lake’s water quality (Juma et al., 2014). Over 60% of the Lake Victoria Basin (LVB) experiences degradation, attributed to diverse land use alterations, such as wetland reclamation. These changes lead to heightened sediment and nutrient loading into the lake through surface runoff, aerial deposition, and river inflow (Nyamweya et al., 2023; Scheren et al., 2000). Several studies have shown that significant amounts of pesticides and agrochemicals have been detected in the water and sediments as a result of agricultural intensification in the region (Getenga et al., 2004; Musa et al., 2011; Osano et al., 2003). Eutrophication of the lake has also been aided by water contamination from municipal and industrial waste and improper solid waste management particularly in the bays and gulfs (Nyenje et al., 2010; Oguttu et al., 2018; Olokotum et al., 2021). Additionally, small-scale gold mining in some parts of the Tanzanian catchment could lead to mercury discharges into the lake water if mining wastes are not properly contained (Campbell et al., 2003).

Environmental changes, particularly those associated with climate conditions such as changes in precipitation, temperature, and hydroclimatic extremes (e.g., floods, droughts, and heatwaves) significantly influence water quality. Numerous studies indicate that weather exacerbates the most severe impacts on water quality (Amanullah et al., 2020; EPA, 2016; Mimikou et al., 2000; Shinhu et al., 2023; Whitehead et al., 2009). Increased rainfall variability impacts drinking water quality significantly leading to notable changes in water quality parameters, often shifting from clear to turbid water (Bastiancich et al., 2022; Turyasingura et al., 2023). It also promotes the prevalence of cyanobacteria, worsening eutrophication and impacting the physical, chemical, and biological parameters, as well as nutrient availability (Ojok et al., 2017). Thus, as land use/land cover (LULC) and climate changes continue to evolve, it is likely that conditions favoring the degradation of water quality will occur more often. Consequently, there is a need to monitor water quality to ensure its sustainability for multiple purposes such as human consumption, agriculture, energy, and biodiversity.

As a transboundary resource, the LVB region has yet to implement the basin-wide regulatory measures, technological breakthroughs, and planning necessary to slow the rate at which the lake’s WQ deteriorates (Semyalo, 2021). Some member states face cost constraints in conducting continuous monitoring and relying solely on field samples may not adequately capture the geographical and temporal diversity necessary for comprehensive lake water quality monitoring and management (Dube et al., 2015). Hence, there is a need to incorporate remote sensing as a useful technology for monitoring WQ parameters (Sent et al., 2021).

Remote sensing (RS) offers significant benefits over traditional methods by providing a comprehensive view of water quality, facilitating improved monitoring of spatial and temporal variations. The growing interest in the usage of RS and satellite data is based on its technological advances, affordability, and good spatio-temporal resolution that permit getting information over wide areas (Mashala et al., 2023). Additionally, it provides access to historical data which allows us to track the changes and patterns of many WQ factors over time (Werdell et al., 2018). It also serves as a valuable resource for planning field surveys and collecting samples, as well as offering reliable assessments of optically active components required to define water quality (Dube et al., 2015). Satellite products are indeed a valuable alternative to in situ measurements; however, their complexity arises, from having distinct underlying assumptions, computation algorithms of parameters, and possible improper spatial and temporal image resolutions (Corbari et al., 2016). That is why it is important to validate the remote sensing data with in situ measurements whenever possible, prior to use in analysis (Wu et al., 2019).

Numerous studies have utilized satellite-based water quality monitoring to supplement in situ data, enabling the assessment of spatial and temporal trends in water quality across both inland and coastal waters. Harvey et al. (2015) compared chlorophyll-a concentrations retrieved from MERIS (Medium Resolution Imaging Spectrophotometer) data with ship-based monitoring during the productive seasons of 2008 and 2010 in a coastal area of the Baltic Sea. Their findings revealed a strong correlation between satellite-derived chlorophyll-a measurements and in situ measurements taken within a short time frame (0–3 days), with an RMSE of 64%. Gohin et al. (2019) examined monthly averages of satellite-derived chlorophyll-a over two periods (1998–2003 and 2012–2017) in the English Channel. Both the in situ and merged satellite (SeaWiFS-MODIS/Aqua-MERIS-VIIRS) chlorophyll-a time series indicated a decrease in chlorophyll-a concentrations in the channel during May, June, and July. Ogashawara and Moreno-Madriñán (2014) created an empirical bio-optical algorithm for the Moderate Resolution Imaging Spectroradiometer (MODIS) daily surface reflectance product to monitor Chl-a in Lake Thonotosassa, USA. The results demonstrated that MODIS products can effectively monitor water quality in small lakes. Ross et al. (2019) also developed AquaSat, a dataset with over 600,000 matchups of water quality measurements and Landsat reflectance data from 1984 to 2019, spanning diverse water bodies across the USA. Their work revealed clear water quality and reflectance relationships. These studies, among others, underscore the increasing reliance on satellite data for comprehensive water quality monitoring at large spatiotemporal scales.

In Lake Victoria, numerous studies have utilized RS and satellite data to evaluate its water quality in various sections (Juma et al., 2014; Mutyaba et al., 2018; Sichangi & Makokha, 2017). Additionally, this data has been instrumental in analyzing the impacts of climate change (Awange et al., 2013), and LULC changes (Kiggundu et al., 2018; Mugo et al., 2020; Onyango et al., 2021) in the LVB. The results all showed increasing trends of these global changes which pose a serious threat to the environment and water quality. The diverse and successful usage of RS data and satellite products to assess water quality and to explore impacts of climate and LULC changes in the LVB shows the potential of utilizing RS to fulfill the objective of this study which is to analyze water quality changes in Lake Victoria by utilizing existing RS products of precipitation, land use and water quality.

While some studies such as Mugo et al. (2020) and Nyamweya et al. (2023) agree that the key drivers of water quality decline in Lake Victoria are climate and land use change, distinct relationships between these changes and the water quality of the lake are yet to be. This study examines global satellite products of chlorophyll-a (Chl-a) and turbidity (TUR) concentrations across the lake over the period (2000–2022), exploring the trends and variability of these concentrations in relation to changes in precipitation and LULC in the LVB. The study places emphasis on two key regions, i.e., the Winam Gulf in Kenya and inner Murchison Bay in Uganda, which experience poor water quality and have undergone major LULC changes in recent times. Additionally, the study validates the satellite products against in situ measurements, thereby enhancing the discussion on the feasibility and accuracy of using these technologies for water quality monitoring.

## Datasets and methods

### Study area

**Fig. 1:**
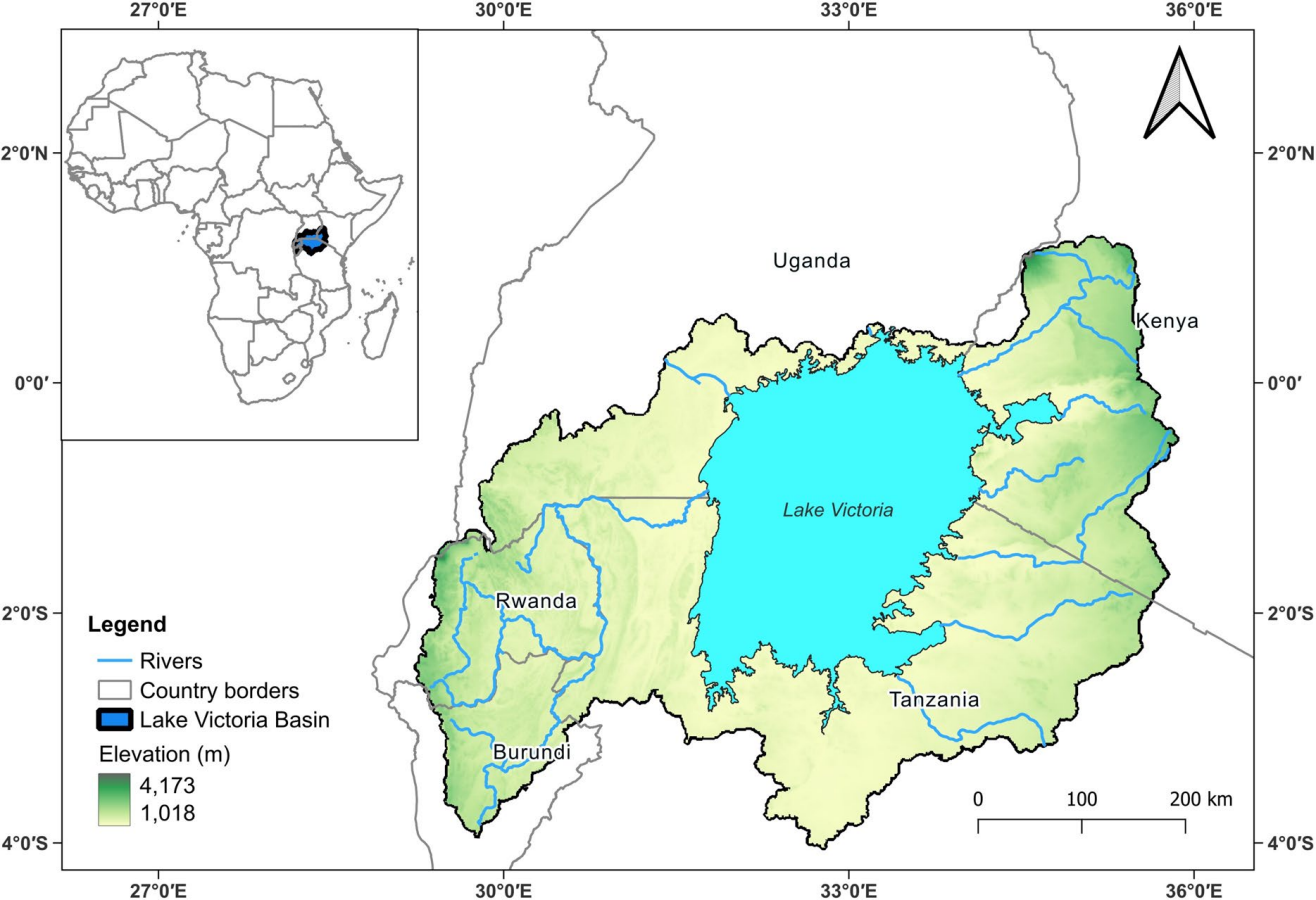
Map of Lake Victoria basin, with major rivers and surface elevation

Lake Victoria, spanning an area of 68,800 km^2^, is shared by three nations (Tanzania 49%, Uganda 45%, and Kenya 6%). Its basin area, however, extends to 194,000 km^2^ spread across five countries (Juma et al., 2014). Its climate ranges from tropical rainforest with year-round rainfall (117 km^3^/year) over the lake to a semi-arid climate with occasional droughts in some parts, and temperatures range from 12 to 26 °C (Miriti, 2022). The LVB experiences rainfall in two distinct seasons: the “long rains” from March to May (MAM) and the “short rains” from October to early December (OND) (Nicholson, 2015). These rainfall patterns are influenced by large-scale forces such as zonal winds over the central Indian Ocean and inter-tropical convergences (Nicholson, 2017). On the one hand, these forces bring substantial rainfall during the MAM and OND periods. On the other hand, the driest months typically occur in June, July, and August. The main vegetation types found throughout the LVB are montane forests, savannahs, grasslands, wetlands, woodlands, and croplands (Odada et al., 2009). The LVB is densely populated, with 300 people per km^2^, growing by 3.5% annually (Marcus, 2022). Major cities such as Kampala and Jinja in the Inner Murchison Bay in Uganda, Kisumu, at the Winam Gulf in Kenya, and Mwanza in Tanzania have expanded, along with the development of new towns along the lake shore (Nyamweya et al., 2020). Figure 1 shows the LVB, its major tributary rivers, and elevation from the Shuttle Radar Topography Mission (SRTM).

### Datasets used in the study

In this study, turbidity and chlorophyll-a were the water quality parameters considered as these can be directly derived from ocean-color satellite remote sensing data. Chlorophyll-a indicates phytoplankton abundance and biomass, reflecting trophic status (Keukelaere & Knaeps, 2021), while turbidity indicates water clarity, affected by factors like river run-off, phytoplankton growth, climate, and watershed changes (Crétaux et al., 2020). Satellites like MODIS, MERIS, Sentinel-2, and Landsat enable accurate analysis of WQ parameters through the connection established between in situ measurements and emitted/reflected radiation in spectral bands such as the green, red, and infrared bands (Ambrose-Igho et al., 2021; Papenfus et al., 2020; Watanabe et al., 2018). Chl-a and TUR are derived from Lake Water-Leaving Reflectance (LWLR), an important indicator of biogeochemical processes and habitats in the water column (Crétaux et al., 2020), using globally validated algorithms (Dogliotti et al., 2015; Keukelaere & Knaeps, 2021). Numerous studies have utilized satellite products to monitor water quality. For instance, Rojamadhuvanthi et al. (2021) estimated chlorophyll-a (Chl-a) in lakes Parakkkai and Therekal using Sentinel-2 satellite imagery. Ahmed et al. (2010) employed semi-empirical algorithms to retrieve turbidity and total suspended matter product from MODIS imagery. Toming et al. (2016) assessed the suitability of Sentinel-2 Multispectral Imager (MSI) data for mapping various lake water quality parameters, including Chl-a, watercolor, colored dissolved organic matter (CDOM), and dissolved organic carbon (DOC). They compared in situ data with band ratio algorithms derived from Sentinel-2 Level-1C and atmospherically corrected Level-2A images, finding good correlations with *R*^2^ values ranging from 0.52 to 0.92.

For this study, two RS WQ products were used, i.e., (1) “ESA” data from the Lakes Project of the European Space Agency Climate Change Initiative (ESA CCI-Lakes—v1.1)) and (2) “Copernicus” data which is a Lake WQ product from Copernicus Global Land Service (CGLS). The Copernicus data (v1.3.0) comprised monthly turbidity and trophic state index (TSI) at a spatial resolution of 300 m derived from the OLCI sensor on board of Sentinel-3. TSI measures phytoplankton productivity and eutrophication. The Copernicus data were obtained from the Copernicus Global Land Service website (https://land.copernicus.eu/global/products/lwq) for the period of 2016–2022. The retrieval algorithms for this dataset are stipulated in Warren et al. (2021). Chl-a was derived from TSI according to the table adapted from (Simis, 2020) shown in Supplementary Material (Table S2). ESA data records of turbidity and chlorophyll-a at a spatial resolution of 100 m and daily temporal resolution were acquired from the ESA website (https://climate.esa.int/en/projects/lakes/data/) from 2000 to 2012 (derived from the MERIS sensor on board ESA’s ENVISAT satellite) and 2016 to 2019 (derived from the OLCI sensor on board of Sentinel-3). The “*Algorithm Theoretical Basis Document*” for this data product, readily available on the website, provides a full explanation of the algorithms and corrections used to create these estimates. Both datasets were already preprocessed and ready for use. Chlorophyll-a estimates were measured in mgm^−3^ whereas turbidity was in NTU.

Past records of in situ measurements of Chl-a and TUR data were collected from the National Water and Sewerage Corporation (NWSC), Uganda, and these were used to validate RS Chl-a and TUR data. The measurements, available irregularly, were gathered monthly from March 2013 to June 2022 at 26 sampling locations in the Inner Murchison Bay (IMB). Approximately 7% of the data was missing, reflecting occasional gaps in the monthly records.

Monthly precipitation records at 0.05° spatial resolution for the period of 2000 to 2022 were retrieved from the CHIRPS (Climate Hazards Group InfraRed Precipitation with Station data) website (https://www.chc.ucsb.edu/data/chirps) and used to analyze changes in rainfall across the LVB over time. These CHIRPS rainfall estimates have been used in several studies of African precipitation even for the LVB and have been determined to be adequate when compared to ground truth data than other rainfall products such as African Rainfall Climatology Version 2 (ARC2), Precipitation Estimation from Remotely Sensed Information using Artificial Neural Networks–Climate Data Record (PERSIANN‐CDR), and TAMSAT African Rainfall Climatology And Timeseries (Das et al., 2022; Diem et al., 2019; Funk et al., 2015).

Annual land cover maps at a spatial resolution of 300 m were obtained from the Land cover dataset from the European Space Agency Climate Change Initiative (ESA-CCI). These are land cover classification gridded maps from 1992 to present derived from satellite observations and acquired from the website (https://www.esa-landcover-cci.org) for the years between 2000 and 2022. The ESA Land Cover dataset is recommended for mapping land cover changes globally over a long period of time and at a relatively fine resolution (Liu et al., 2018; Plummer et al., 2017). The dataset has an overall weighted-area accuracy of 73% based on 3167 samples, but it has some limitations due to varying quality in certain regions, such as parts of Amazonia where MERIS FRS data coverage is limited, which can affect the land cover map (Defourny et al., 2009). Using the LC Classification System developed by the FAO, the land cover maps are presented with 37 original LC types (Santoro et al., 2017). In addition, Landsat 8 images were downloaded from the website (https://earthexplorer.usgs.gov/) to be used in the computation of the Normalized Difference Vegetation Index (NDVI) for the Winam Gulf and Inner Murchison Bay to further aid in the assessment of vegetation across the different seasons of the year. This was done for 2016 and 2021.

### Comparison of global satellite products with in situ measurements

We compared the satellite products for water quality with past in situ measurements to assess the extent to which they aligned. WQ parameters from the 26 sampling locations in the IMB were averaged at a monthly scale and compared with the ESA and Copernicus satellite data. The sampling locations can be seen in Fig. S1(Supplementary Material). The RS Chl-a and TUR raster files were realigned and resampled using the nearest neighbor method in QGIS to the same pixel size (0.00833°) at a monthly timestep. Statistical metrics, such as mean, median, standard deviation, correlation coefficients, time series, and graphical criteria (e.g., histograms, box plots), were used to compare the RS and in situ data. The evaluation aimed at assessing the accuracy and correlation between in situ and RS data. The mathematical models for the statistical metrics utilized are shown in the supplementary material (Table S1).

### Assessment of water quality in the lake

A visual assessment of maps and time series was also carried out using processed satellite data of chlorophyll-a and turbidity to assess the ecological status of the lake from 2005 to 2022. The analysis involved both ESA and Copernicus data due to the challenges of missing data and the limited timeframe of the satellite data available. Pollution hotspots, notably the Winam Gulf and the Inner Murchison Bay, were identified across the lake, prompting a detailed study to investigate the links between land use changes, precipitation patterns, and water quality variations in these regions.

### Spatial and temporal variability of rainfall

This analysis involved the use of monthly mean CHIRPS precipitation raster files spanning from 2000 to 2022. The mean annual precipitation and coefficient of variation (CV) over LVB were computed. The CV was computed as the ratio of the standard deviation to the mean and was used to classify the degree of variability of rainfall events as less (CV < 20), moderate (20 < CV < 30), and high (CV > 30) (Nkwasa et al., 2022). The formulas are shown in Table S1, supplementary material.

The Mann–Kendall (MK) (Kendall, 1975; Mann, 1945) test was then applied to the data to identify trends. This test has previously been used to analyze temporal trends of climatic variables such as precipitation and temperature (Mallick et al., 2021; Wang, 2018; Wang et al., 2012). Since the test is nonparametric, the data does not have to adhere to a normal distribution. However, it does presuppose that there is no autocorrelation in the time series. Typically, trends are considered significant when they achieve a 95% confidence level (Buo et al., 2021). The magnitude of the trends was also calculated using the nonparametric Theil-Sen estimator (Sen, 1968) which is computed by taking the median of the slopes between each pair of points in the time series data.

### Land use/cover change analysis

The nomenclature of the land cover maps was reclassified from the 36 original classes in the LVB to 8 major land classes, i.e., agriculture, forest, grassland, wetland, built-up, sparse vegetation, bare area, and open water as shown in Table S3, Supplementary Material (Mousivand & Arsanjani, 2019). This was done to accommodate classes that are relevant to the study area and represent specific land use changes related to ongoing human activities.

To visually depict LULC changes over time, maps were created to highlight areas that experienced growth and those that remained unchanged. Using the GIS vector geoprocessing tool, land cover shapefiles from two time periods (e.g., 2000–2010, 2010–2020, or 2000–2020) were intersected to identify classes that showed no changes in each area. This intersection indicated the absence of change in the land cover class. The intersected land cover classes were then reclassified as “no change” in the resulting map, which represented the “no change” classes and newly gained areas from the recent land cover map, e.g., for 2010 for the period of 2000–2010.

#### LULC change matrices

Land cover change matrices are used to analyze how different land cover areas have changed over time. This involves comparing maps of the same location from two distinct time periods and generating a cross-tabulation matrix. The matrix shows the area that has changed between different land cover categories. Diagonal entries indicate land persistence, while off-diagonal entries indicate land cover change (Aldwaik & Pontius, 2012). Transition matrices have been widely used in landscape ecology and land use/cover change studies (Han et al., 2009; Romero-Ruiz et al., 2012; Takada et al., 2010).

Three levels of analysis exist, i.e., interval, categorical, and transition levels. The interval level examines changes between two time periods, the categorical level assesses the intensity of transformation between categories, and the transition level focuses on the dynamics and intensity of transitions within a category relative to others. Annual change intensities are computed at the interval level, while the magnitude and intensity of gross gains and losses are evaluated at the category level. The transition level investigates changes in categories and their variations and identifies frequently targeted or avoided categories. These analyses compare observed intensities to uniform measures of transition (Aldwaik & Pontius, 2012; Alo & Pontius, 2008). Areal percentage changes for 3 distinct time periods, 2000–2010, 2010–2020, and 2000–2020, were calculated using Eq. 1. Transition matrices were then generated using the Semi-Automatic Classification Plugin in QGIS (Congedo, 2016).

1 $$\text{Percentage change in a class} \, \text{=}\left(\frac{\text{Total class area in a recent year}}{\text{Total class area in the older year}}\times {100}\right)-{100}$$

#### Change budget and intensity analysis

The analysis of relative gain, loss, and persistence among different land cover classes offers deeper insights into the dynamics of LULC changes. To assess the change budget, transition matrices were employed to calculate the uniform gain, loss, and persistence of various land cover classes using the following equations.

2 $$\text{Uniform Gain} \, \text{=} \, {100} \, \text{* }\left( \frac{\text{Column total}}{\text{N}}/\text{Diagonal of each class(unchanged)}\right)$$

3 $$\text{Uniform Loss} \, \text{=} \, {100} \, \text{* }\left(\frac{\text{Row total}}{\text{N}}/\text{Diagonal of each class(unchanged)} \, \right)$$

4 $${\text{Persistence}} \, = \text{ } \frac{\text{Diagonal of each class}\left({\text{unchanged}}\right) \, \text{*} \, {100}}{\text{Row total + Column total}}$$

To conduct the intensity analysis, the average values of gains and losses were calculated and then employed to distinguish between active and dormant changes. An active category change is identified when the intensity surpasses the uniform line, indicating a relatively rapid change. However, a dormant change is observed when the intensity falls below the uniform line, indicating a relatively slow change (Huang et al., 2012).

### Linking impacts of changes in climate and LULC to changes in lake water quality

**Fig. 2:**
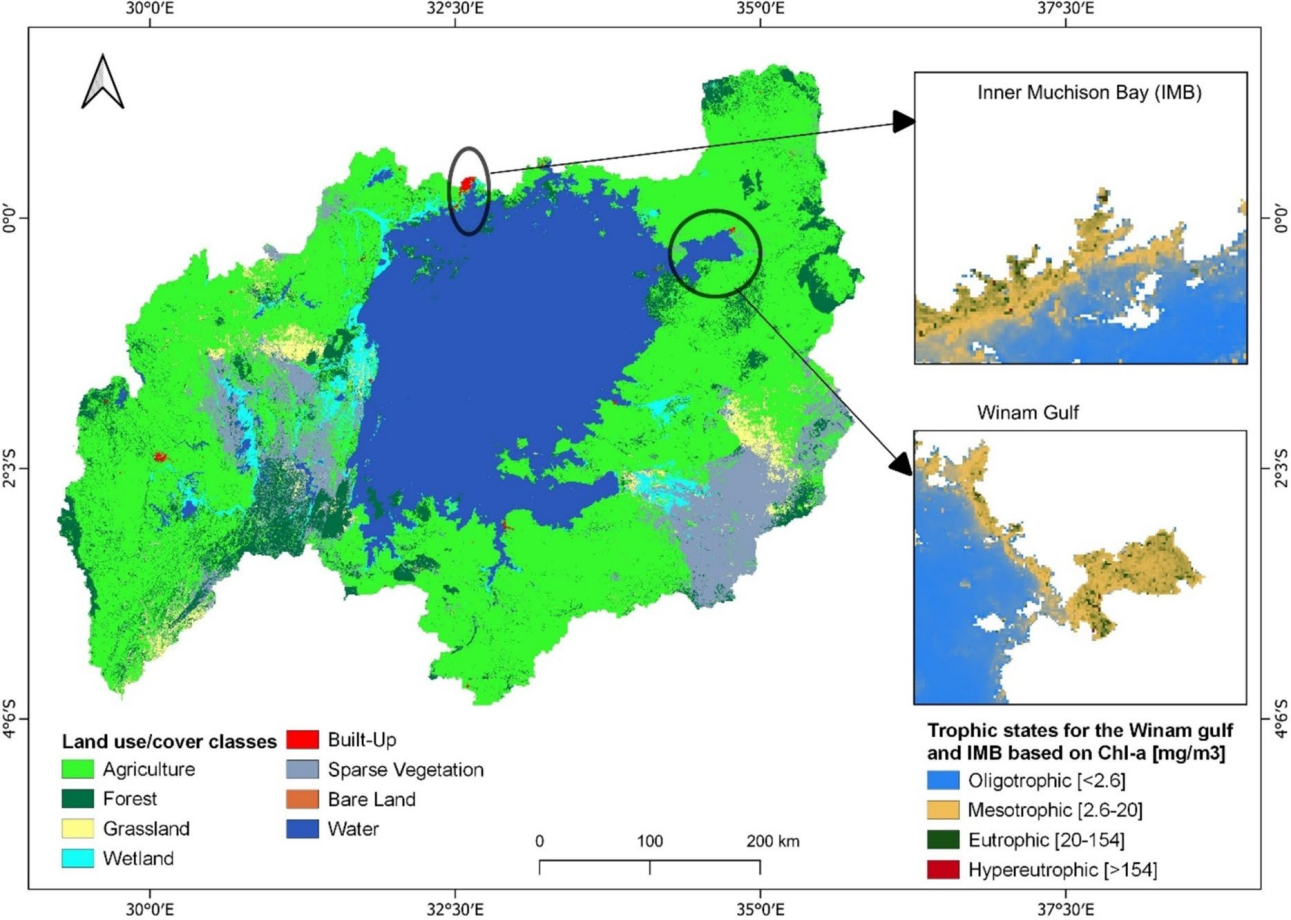
2010 annual average land use/cover classification and trophic state mapping of Winam Gulf and Inner Murchison Bay (IMB) in the Lake Victoria basin based on Chl-a concentrations

To assess the effects of climate and land use/cover changes on water quality, Chl-a, and TUR time series data were extracted from prominent pollution hotspots in Lake Victoria, namely the IMB in Uganda and the Winam Gulf in Kenya. These areas are both ecologically important, pollution-prone (as seen in the 2010 annual average chlorophyll-a map for the lake in Fig. 2), and subject to land use and precipitation changes, making them ideal for studying the relationship between these factors and water quality (Calamari et al., 2006; Kabenge et al., 2016). They also serve as major sources for water abstraction for local communities and major cities like Kampala and Kisumu (Olokotum et al., 2020). The extracted data was plotted and analyzed to understand the trends and variability in water quality parameters and to relate them to climate and land use changes.

## Results

### Comparison of global satellite water quality products with in situ data

**Fig. 3:**
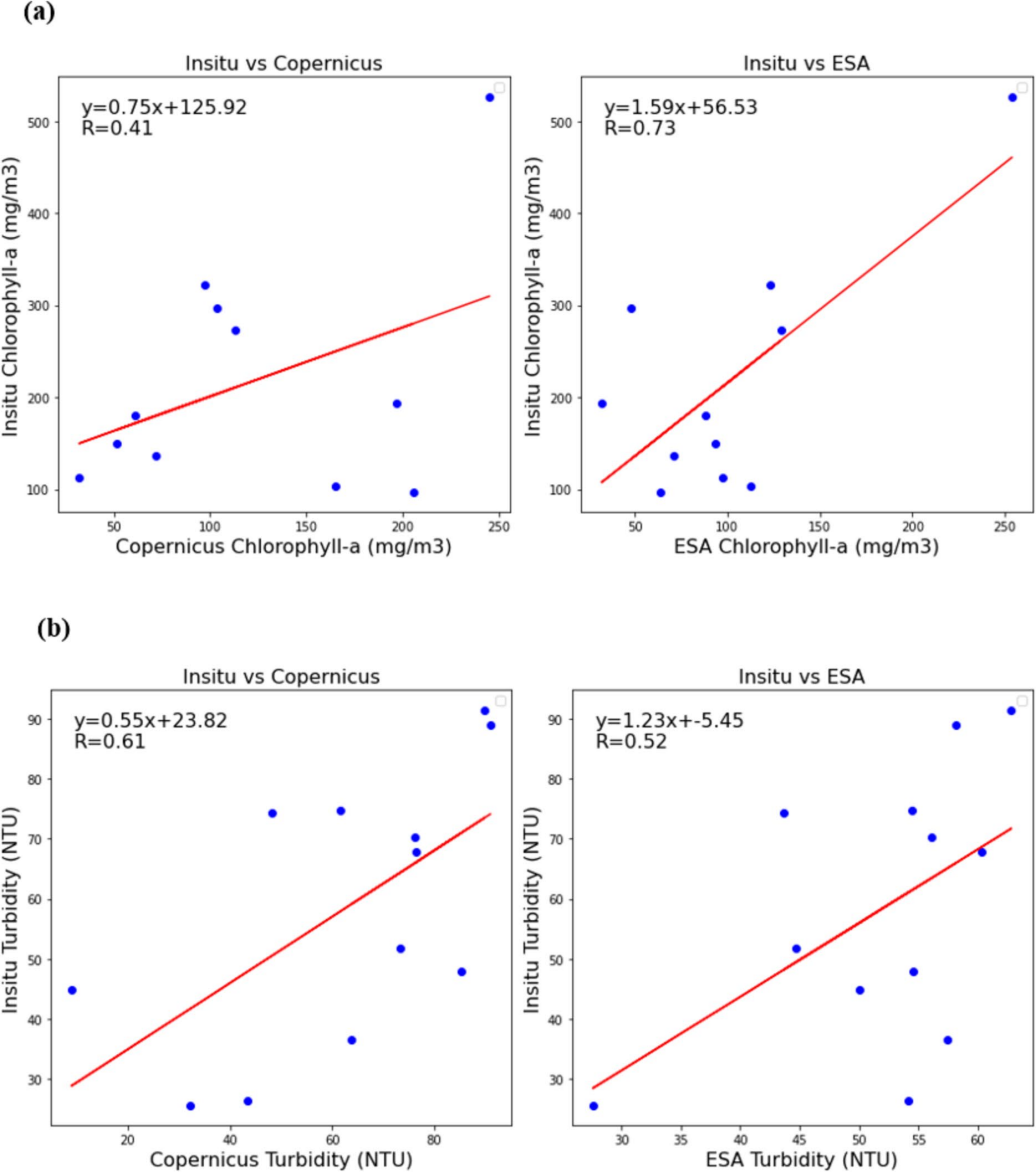
Pearson correlation between monthly in situ, Copernicus, and ESA chlorophyll-a (**a**) and turbidity (**b**) in 2018 at sampling locations in Inner Murchison Bay

The statistical metrics in Table 1 and Fig. 3 compare in situ and satellite data of chlorophyll-a and turbidity in the Inner Murchison Bay (IMB) for 2018. Additionally, Fig. S2 in the Supplementary Material presents histograms and boxplots illustrating the distribution of Chl-a and TUR concentrations for In-situ, Copernicus, and ESA data in the IMB for the same year.

**Table 1:** Statistical comparison of the WQ parameters for Copernicus and ESA data for 2018

Statistical metric	Chlorophyll-a (mg/m^3^)		
	In situ	Copernicus	ESA
Mean	217.55	122.05	101.17
Standard deviation	129.47	70.63	59.03
25% (first quartile, Q_1_)	124.26	66.24	67.49
50% (median, Q_2_)	179.79	103.84	93.69
75% (third quartile, Q_3_)	285.00	181.02	118.24
Pearson correlation		0.41	0.73
*p*-value at 0.05 alpha		0.21	0.01
Statistical significance		False	True
	Turbidity (NTU)		
	In situ	Copernicus	ESA
Mean	58.41	62.52	52.18
Standard deviation	22.78	25.07	10.04
25% (first quartile, Q_1_)	42.77	47.09	48.68
50% (median, Q_2_)	59.82	68.49	54.53
75% (third quartile, Q_3_)	74.51	78.69	57.61
Pearson correlation		0.61	0.52
*p*-value at 0.05 alpha		0.03	0.08
Statistical significance		True	False

The in situ Chl-a data showed the highest standard deviation, indicating more variability that was not captured in the Copernicus and ESA data. The Pearson correlation between in situ and Copernicus at 0.41 with a *p*-value of 0.211 indicated a moderate positive correlation that was not statistically significant. This suggested that Copernicus data may not reliably estimate in situ chlorophyll-a levels. However, the in situ and ESA data correlation was 0.73 (*p* = 0.011), indicating a strong and significant positive correlation, making ESA a more reliable source for estimating chlorophyll-a concentrations in the study area.

For TUR, the standard deviations indicated that both in situ and Copernicus data exhibited higher variability compared to ESA data, suggesting that Copernicus data more accurately reproduced the in situ measurements than the ESA data. The Pearson correlation coefficient between in situ and Copernicus was 0.61, indicating a moderate positive correlation that was statistically significant with a *p*-value of 0.036 suggesting that Copernicus measurements can be considered fairly reliable in estimating in situ turbidity levels. In contrast, the correlation between in situ and ESA was slightly weaker at 0.52 and was not statistically significant with a *p*-value of 0.085, indicating that ESA measurements might not consistently align with in situ data or that more data might be needed for a reliable correlation.

The datasets were visually compared with in situ data across varying time spans (Fig. S3, Supplementary Material), contingent upon data availability, specifically Copernicus (2016 to 2022) and ESA (2016 to 2019). Chl-a values from Copernicus were generally underestimated compared to the in situ measurements, while the turbidity values showed fewer underestimations. Overall, graphically, TUR had a better fit than Chl-a with Copernicus data having similar trends at most times than ESA data. Based on this analysis, the water quality analysis for Chl-a was done using ESA data, while that of TUR with Copernicus data.

### Lake Victoria water quality analysis

**Fig. 4:**
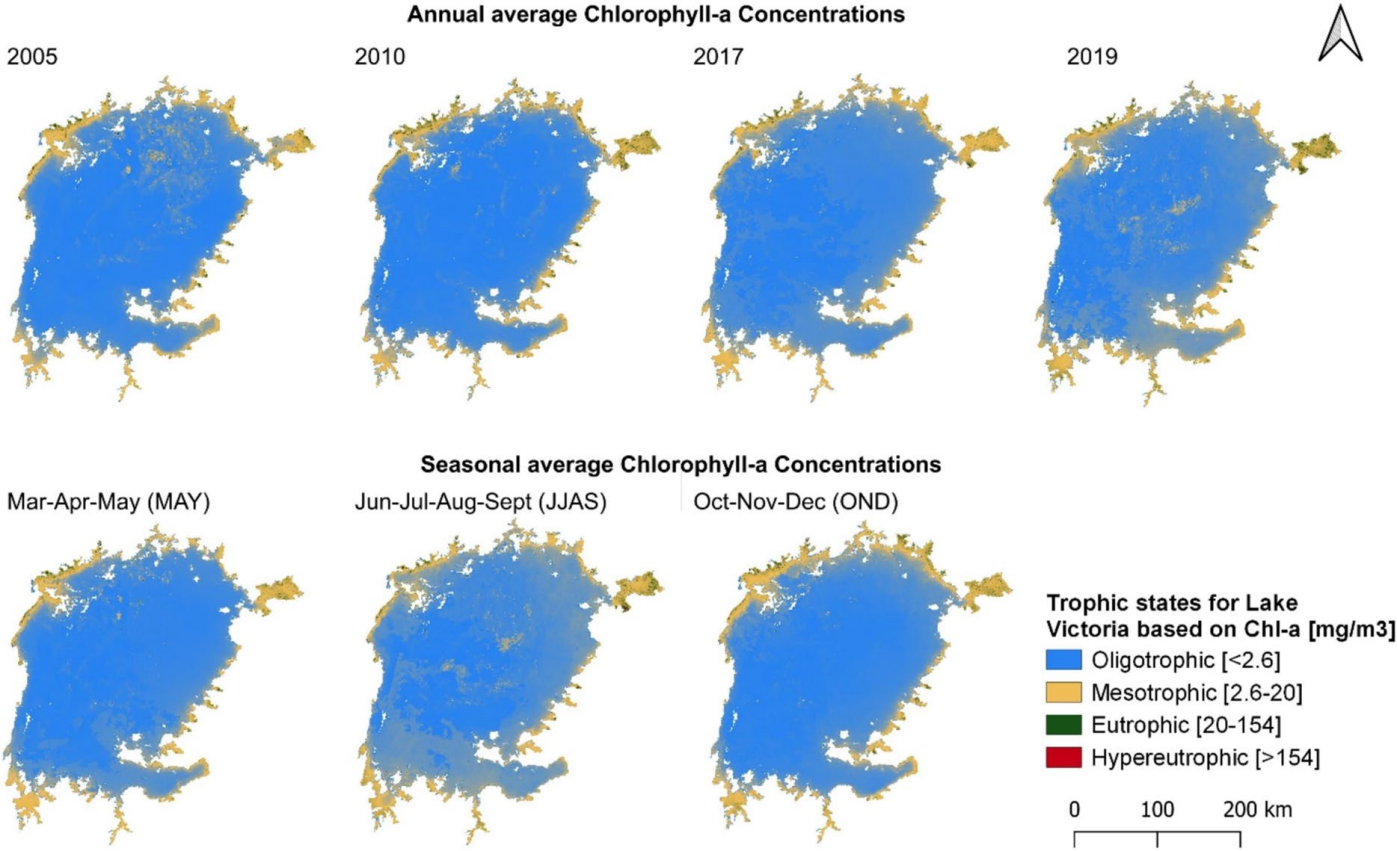
Trophic states for Lake Victoria for four different years based on mean chlorophyll-a concentrations and seasonal averages between 2016 and 2019 using ESA data

Figure 4 displays the spatial distribution of chlorophyll-a using ESA data categorized according to trophic levels (Table S2, Supplementary Material) adapted from Simis (2020). Most of the lake, particularly the inner waters, falls under the oligotrophic category, indicating good water quality. However, the shores consistently exhibit eutrophic-mesotrophic conditions throughout the years. In 2010 and 2019, highly eutrophic conditions were observed, particularly in the Winam Gulf. Seasonal water quality analysis reveals no significant differences between the MAM and OND rainy seasons. However, the Kenyan shores near the Winam Gulf experienced higher eutrophic conditions during the OND, likely due to runoff from nearby farmlands and urban areas. During the dry JJAS season, similar eutrophic conditions were observed, particularly in the Winam Gulf.

**Fig. 5:**
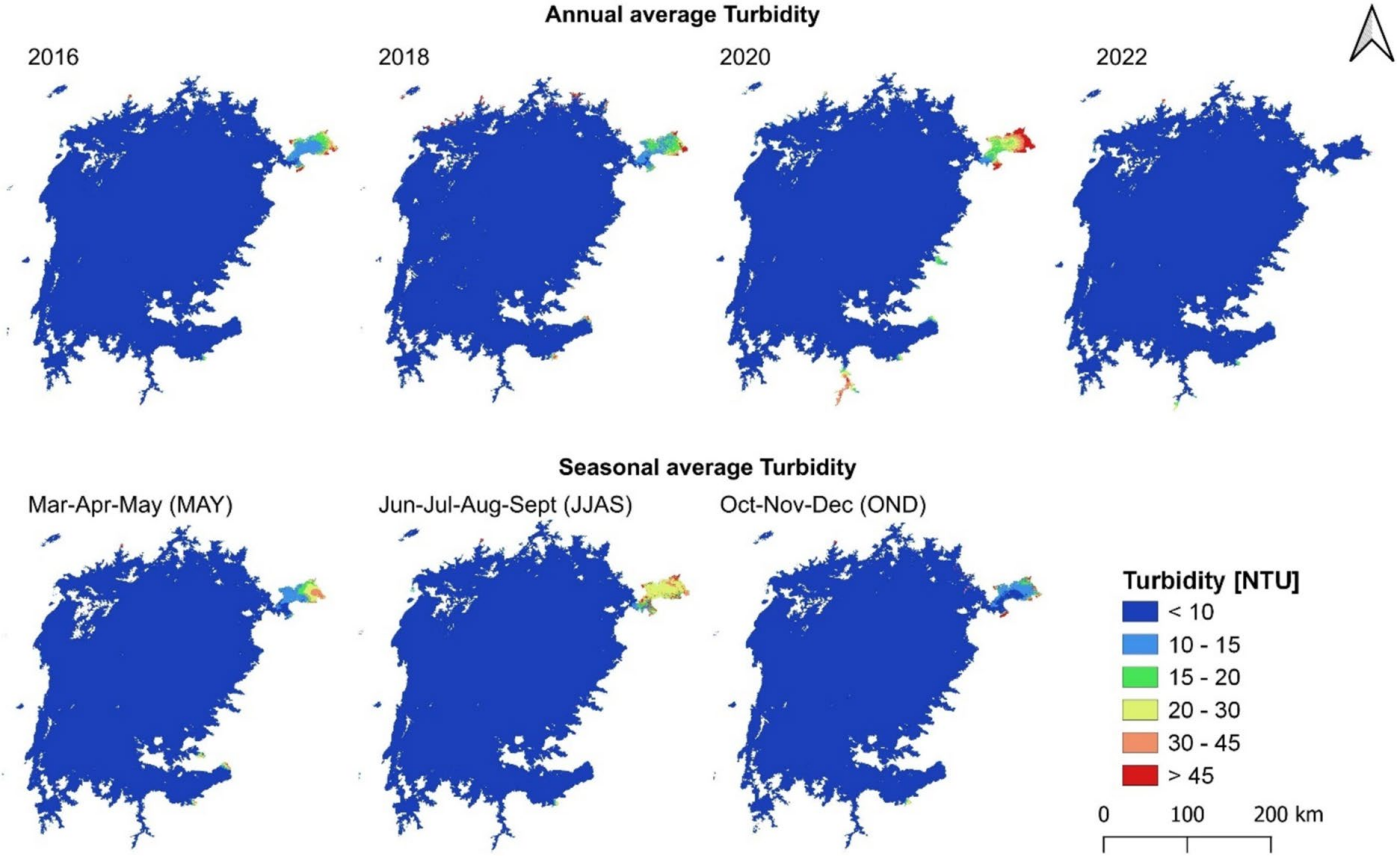
Mean turbidity in Lake Victoria for four different years and seasonal averages between 2016 and 2019 using Copernicus data

Figure 5 illustrates turbidity distribution across Lake Victoria from 2016 to 2022 (the even years) using Copernicus data. Most of the lake exhibited low turbidity levels, below 10 NTU. The Winam Gulf in Kenya had the highest turbidity, peaking in 2020. This increase, particularly in the Winam Gulf and some southern shores, can be attributed to heavy rains that transported more sediments into the lake. By 2022, turbidity improved, with values below 10 NTU throughout the lake. Seasonally, similar patterns to Chl-a were observed, with minimal differences across the lake except in hotspots like the Winam Gulf, where turbidity reached 20–30 NTU during the dry JJAS season and improved during the wet OND season.

In summary, the lake’s overall water quality has remained relatively stable, particularly in the inner waters. Nevertheless, certain areas, such as the shores and the Winam Gulf, displayed significant trends that need to be examined further.

### Precipitation analysis

**Fig. 6:**
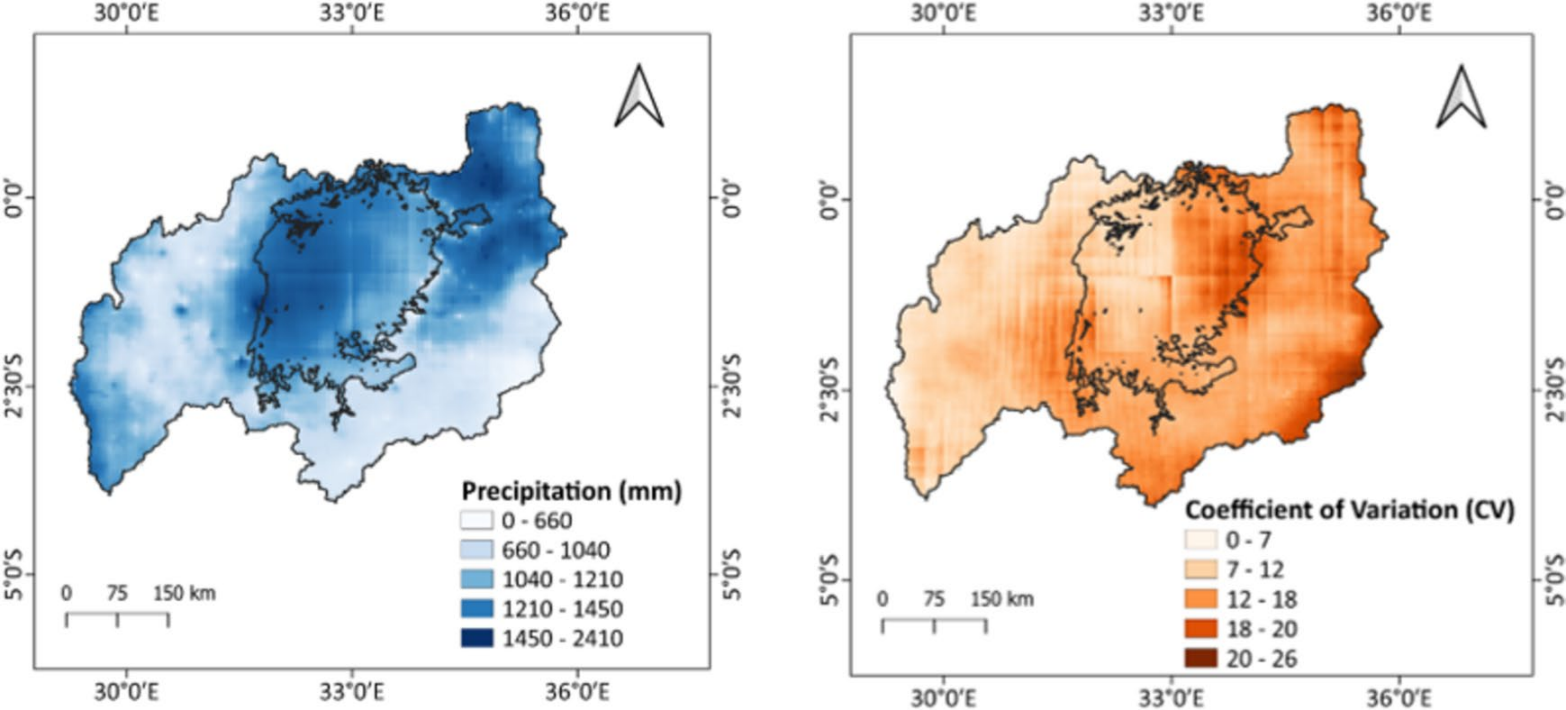
Mean annual precipitation(left) and CV of precipitation over the LVB for the period 2000–2022

The mean annual precipitation was computed as the mean of the yearly totals over the time frame of 23 years. From Fig. 6, the minimum amount of rainfall received was 658 mm whereas the maximum was 2409 mm experienced in the northeastern part of the basin which showed a high spatial variability across the basin.

The coefficient of variation (CV) reflects the interannual variability of annual precipitation as shown in Fig. 6. The moderate fluctuations (CV > 20) were found in areas with the lowest mean annual precipitation, while the lowest variations (CV < 20) were found in regions with the highest annual precipitation. This suggests that water availability is more unpredictable in regions with low yearly precipitation due to high variances around the low precipitation.

The monthly mean rainfall across the LVB ranges from 35 mm in July to 202 mm in April depicting the two rainy seasons with the “long rains” between March and May and the “short rains” between October and December as shown in Table 2. The mean annual rainfall averaged over the catchment was 1273 mm for the period 2000–2022 with the highest rainfall received in 2020 (Fig. S4, Supplementary Material).

**Table 2:** Monthly mean precipitation (mm) over the LVB for the period 2000–2022

Jan	Feb	Mar	Apr	May	June	July	Aug	Sept	Oct	Nov	Dec
86	81	149	202	141	49	35	60	77	117	146	128

Trend analysis was conducted using the Mann–Kendall test at a monthly scale to assess seasonal trends. The trends were depicted with a range of − 15 to 25 mm per year. It was observed that in the first wet season (MAM), there was a generally decreasing trend, with March showing the strongest decline. In the second wet season (OND), the trend started with a decrease and then transitioned to an increase towards the end. During the dry season (late June to September), there was an overall increasing trend across the basin, but with a decrease in the upper part. Notably, there was a clear spatial pattern in the Ugandan part of the LVB, with a significant decreasing trend in the first wet season and an increasing trend in the second wet season. The Sen’s slope parameter was also computed, and it showed where and when precipitation changed the most in mm/year. A decreasing trend during the first wet season and an increasing trend at the start of the dry season were observed. Majorly, there was no trend in November which then shifted to an increasing trend in December. The complete overview of results from these tests can be found in Fig. S5 and Fig. S6, Supplementary Material.

### LULC change analysis

Table 3 presents the percentage changes in land cover class areas over time. Positive values indicate an expansion in coverage, while negative values indicate a reduction. Built-up areas had the highest increment in coverage at 300% between 2000 and 2020. On the other hand, bare land suffered the largest loss, at approximately 50%, mostly occurring between 2010 and 2020. The increase in forest cover between 2010 and 2020 can be attributed to reafforestation efforts and government policies promoting forest conservation in the basin.

**Table 3:** Percentage change in area for the different time periods

Land cover classes	Percentage change in area		
	2000–2010	2010–2020	2000–2020
Agriculture	0.75	− 0.85	− 0.11
Forest	− 3.50	4.85	1.18
Grassland	− 2.10	− 1.21	− 3.28
Wetland	− 0.34	− 0.63	− 0.97
Built-up	136.18	69.28	299.81
Sparse vegetation	− 1.05	− 0.97	− 2.01
Bare land	− 2.16	− 48.78	− 49.89
Water body	0.02	0.01	0.03

**Fig. 7:**
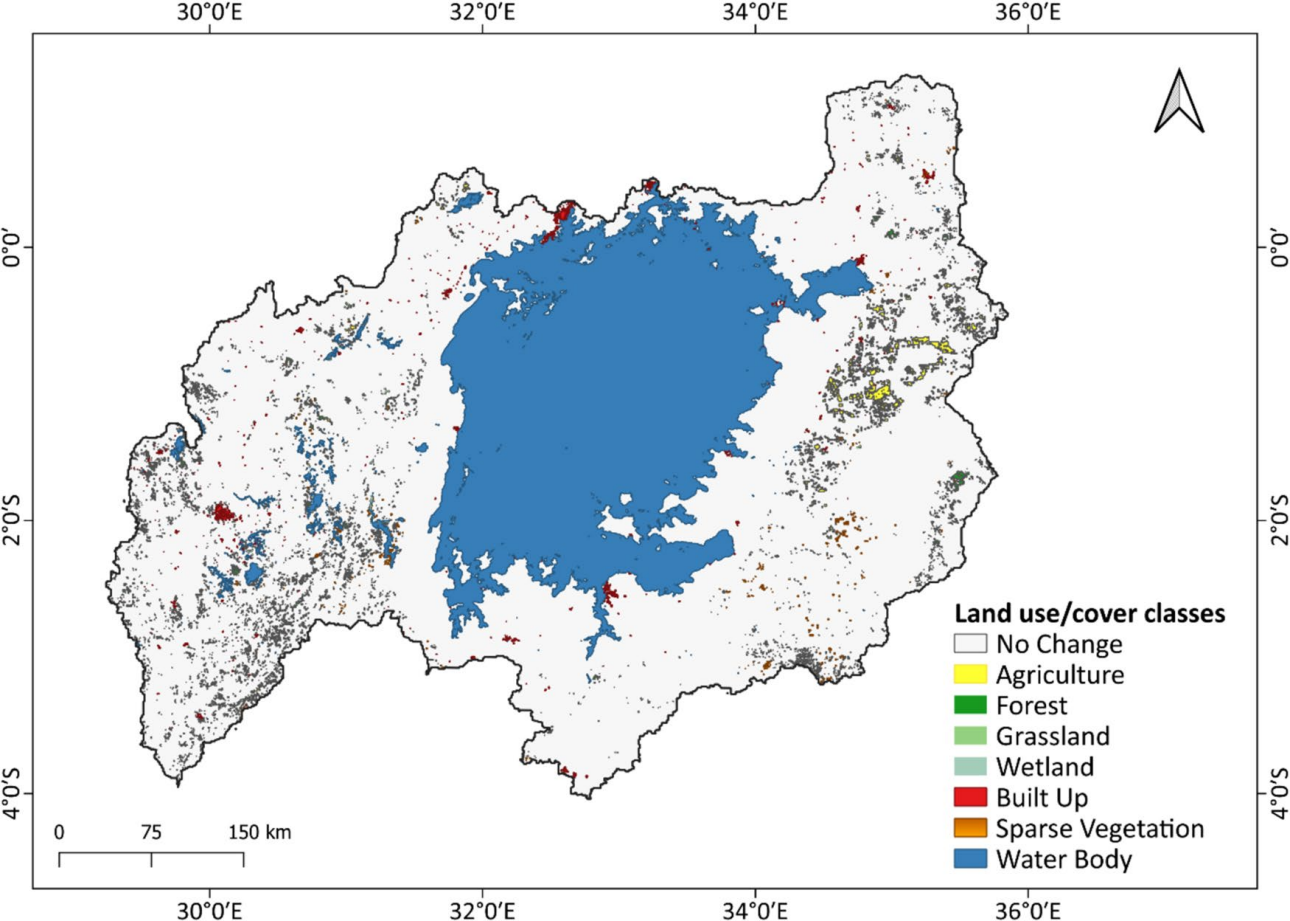
Land cover classes that gained area in the period 2000–2020 in the LVB

Transition matrices were generated to further analyze the LULC changes over different time periods (Table S4, Supplementary Materials). Similar findings were obtained, such as built-up areas having the highest uniform gain at 50% whereas all the other classes gained about 13% between 2000 and 2020. Bare land and water suffered the highest uniform losses at 25% and 27%, respectively. The uniform gain in built-up areas was higher in the first decade, reaching 30%. Overall, the transition matrices demonstrated conservative changes in most of the classes across all time periods, with similar uniform losses and gains observed, except for built-up areas and bare land. Results from the change budget analysis (Fig. S7) also showed that built-up areas experienced the greatest gain, while bare land encountered the highest loss during the analyzed period. The intensity of transitions for the different land cover classes between 2000 and 2020 was also analyzed (Fig. S8). The average loss and gain of the different classes determined the uniform loss and gain at 17% and 14%, respectively. Only the gain of built-up areas can be termed as active as its intensity of transition was above the uniform line. The rest were dormant gains. For the losses, bare land suffered an active loss whereas the rest were dormant. Bare land was predominantly converted into built-up areas, as indicated by the transition matrices, with approximately 4 km^2^ undergoing this transformation between 2010 and 2020. Land cover maps of the Lake Victoria basin from 2000 to 2020 at a 5-year time step (Fig. S9) showed minimal widespread changes in the basin. Figure 7 displays the gain in the land cover changes in the LVB between 2000 and 2020, indicating the persistence of most classes. Agricultural areas increased in Kenya, while built-up areas expanded in the Winam Gulf and along the shores of the lake in Uganda (Kampala and Wakiso districts).

### Linking changes in precipitation and land use/cover to water quality

#### Winam Gulf in Kenya

Table 4 presents the mean annual rainfall, Chl-a, and TUR concentrations from RS data, along with the percentage changes in the areal coverage of different land cover classes in the Winam Gulf, using the year 2000 as the benchmark. The gulf spans a total area of approximately 7918 km^2^. It is important to note that Chl-a concentrations are derived from ESA satellite data, while TUR concentrations are sourced from Copernicus data.

**Table 4:** Mean annual rainfall, Chl-a, and TUR concentrations from RS data, LULC changes over the years in the Winam Gulf

Chlorophyll-a									
Year	2005		2010		2017		2019		
Rainfall (mm)	1177		1519		1513		1787		
Chl-a (mg/m^3^)	23.94		40.87		30.21		50.55		
Turbidity									
Year	2016		2018		2020		2022		
Rainfall (mm)	1235		1640		2255		1909		
TUR (NTU)	14.15		15.92		26.40		3.19		
Percentage increment in area (%) starting from 2000									
Land cover classes	2005	2010	2015	2016	2017	2018	2019	2020	2022
Agricultural areas	1.3	0.5	− 0.1	0	0	0	− 0.1	− 0.1	− 0.2
Forest	− 8.0	− 4.0	− 0.2	0	0	0.1	0.1	− 0.1	0.4
Grassland	− 52.0	− 8.3	− 4.5	0	0	0	0.0	− 4.8	0
Wetland	0.3	− 0.2	− 0.2	142.5	0	0	4.9	− 60.7	153.2
Built-up areas	237.3	49.4	29.2	86.3	0	0	− 0.2	− 38.8	62.8
Sparse vegetation	− 0.9	− 0.9	− 0.5	− 77.7	0	0	0.0	368.5	− 71.7
Bare land	50.0	− 33.3	0	0	0	0	0	0	0
Water body	0	0	0	0	0	0	0	0	0

**Fig. 8:**
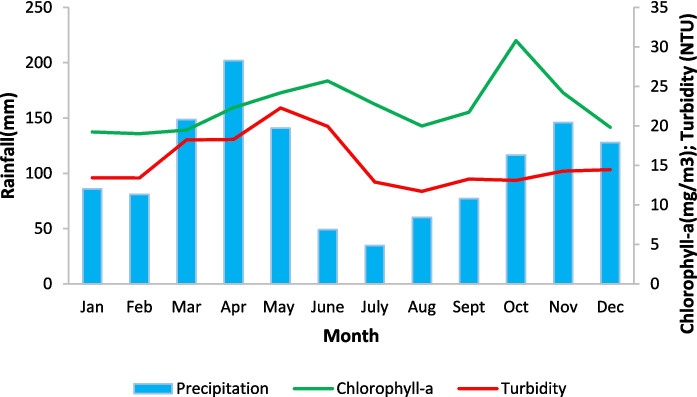
Seasonal variations of precipitation, Chl-a, and TUR from RS data for 2017–2019 in the Winam Gulf

Increased rainfall generally correlates with higher Chl-a concentrations. From 2005 to 2010, rainfall and Chl-a both rose significantly. Between 2010 and 2017, rainfall remained nearly constant while Chl-a decreased, suggesting other influencing factors. From 2017 to 2019, both variables increased, reinforcing their correlation. For TUR, from 2016 to 2020, increased rainfall correlated with higher turbidity, but from 2020 to 2022, turbidity sharply decreased despite significant rainfall, indicating other factors may be affecting turbidity. However, despite TUR’s improvement, the gulf remained mesotrophic-eutrophic for Chl-a. Figure 8 shows the average seasonal variations of turbidity and chlorophyll-a derived from RS data with precipitation for 2017, 2018, and 2019. These years were chosen to compare trends for Chl-a and TUR. Chl-a increases during the first wet season, decreases during the dry season, and peaks again at the start of the second wet season. TUR shows a 1-month lag between its peak in May and the peak of Chl-a in June, followed by a decrease and finally a slow increase during the OND season.

To compare the LULC changes in the Gulf, we examined various land cover classes and their areal gains and losses between 2000 and 2020. Bare land areas experienced the most significant transformation, converting largely into built-up areas, particularly between 2005 and 2010. The built-up areas saw a substantial increase overall, especially from 2000 to 2005, followed by a decline between 2019 and 2020, likely due to an increase in sparse vegetation. In contrast, agricultural areas showed negligible fluctuations in their extent throughout the entire period. We further investigated the use of NDVI to assess seasonal vegetation coverage changes, specifically comparing the years 2016 and 2021 (Fig. S10, Supplementary Material). The analysis revealed an increase in vegetation coverage around the gulf between 2016 and 2021, likely attributed to higher rainfall between 2019 and 2020, which supported agricultural activities. Notably, vegetation density was greater during the MAM season compared to the OND season in both years.

#### Inner Murchison Bay in Uganda

Table 5 shows the mean annual rainfall together with the Chl-a and TUR concentrations in the IMB obtained from the satellite data products with Chl-a from ESA and TUR from the Copernicus dataset. It also shows the change in area as a percentage for the various land cover classes in the IMB starting from 2000. The area of interest (IMB) is on average about 155 km^2^.

**Table 5:** Mean annual rainfall, Chl-a, and TUR concentrations from RS data and LULC changes over the years in the IMB

Chlorophyll-a									
Year	2005		2010		2017		2019		
Rainfall (mm)	1191		1386		1254		1754		
Chl-a (mg/m^3^)	66.61		65.95		160.07		136.62		
Turbidity									
Year	2016		2018		2020		2022		
Rainfall (mm)	1173		1523		1764		1538		
TUR (NTU)	22.92		62.52		12.96		18.54		
Percentage increment in area (%) starting from 2000									
Land cover classes	2005	2010	2015	2016	2017	2018	2019	2020	2022
Agricultural areas	− 34.0	− 17.8	− 8.6	0.2	− 0.9	0	0	− 13.7	− 2.9
Forest	− 41.8	− 12.6	− 3.3	0	0	0	0	− 1.7	0.0
Grassland	0	0	0	0	0	0	0	0	0
Wetland	− 0.8	− 0.8	− 0.4	0	0	0	0	− 0.4	− 0.4
Built-up areas	68.0	12.8	4.2	0	0.3	0	0	5.4	1.1
Sparse vegetation	0	0	0	0	0	0	0	0	0
Bare land	0	0	0	0	0	0	0	0	0
Water body	0	− 0.2	0	0	0	0	0	0	0

Chl-a concentrations ranged between 60 and 160 mg/m^3^, indicating eutrophic to hypereutrophic conditions primarily attributed to pollution from nearby towns and agricultural areas, with the highest concentration recorded in 2017. No clear correlation was observed between annual rainfall totals and Chl-a concentrations in the IMB. From 2016 to 2018, increased rainfall correlated with a sharp rise in turbidity. However, from 2018 to 2020, despite more rainfall, turbidity dropped significantly, suggesting other influencing factors. From 2020 to 2022, as rainfall decreased slightly, turbidity increased, further indicating that factors beyond rainfall affect turbidity levels. Although TUR ranged below 20 NTU from 2020, the bay still experienced eutrophic to hypereutrophic conditions based on Chl-a levels.

LULC changes revealed a significant increase in built-up areas, particularly from 2000 to 2005, while agricultural areas decreased. The reduction in agricultural land use may have contributed to the decreased TUR concentrations after 2020. Additionally, a continuous decline in wetlands and forests, especially between 2000 and 2015, in favor of built-up areas likely increased turbidity in the bay due to higher sediment runoff and erosion. Due to the unavailability of Landsat 8 images during the dry season, NDVI calculations were limited to the wet seasons for the years 2016 and 2021 (Fig. S11, Supplementary Material). In 2016, the MAM season exhibited slightly denser vegetation compared to the OND season. However, in 2021, the OND season showed slightly denser vegetation than the MAM season.

**Fig. 9:**
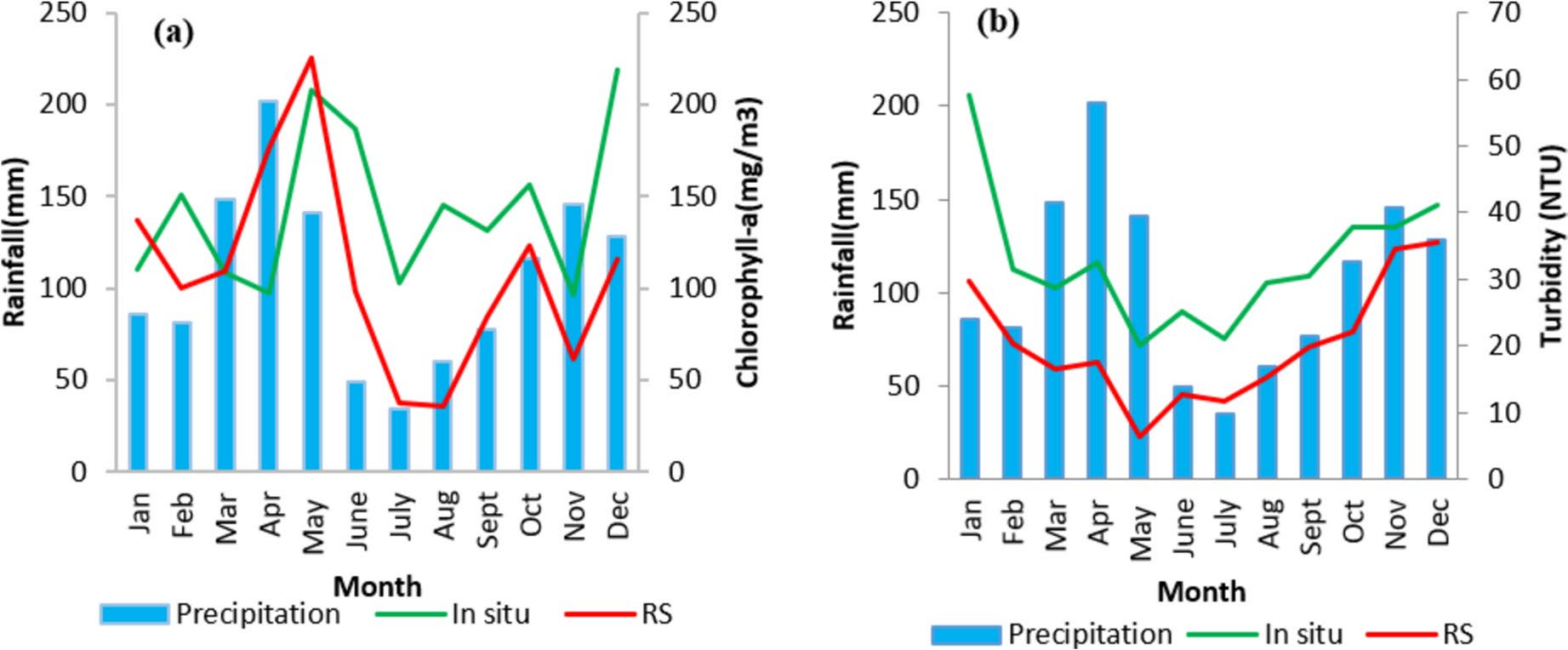
Seasonal variations of Chl-a (**a**) and TUR(**b**) in the IMB

Figure 9 illustrates the seasonal variations of Chl-a and TUR in the IMB for 2017, 2018, and 2019. Based on the ESA RS product, Chl-a levels increased at the start of the MAM season, peaking in May, and then declined during the dry season. Chl-a levels began to rise again from August to October, experienced a sharp decline in November, and increased in December. When compared with in situ Chl-a data, similar trends were observed during the OND season. However, for the MAM season, there was a 1-month lag between March and April, with a decrease at the start of the MAM season and another decline in September. Overall, the RS product underestimated in situ Chl-a levels. Chl-a exhibited a moderate relationship with rainfall in the IMB, with in situ measurements generally increasing after periods of high rainfall, suggesting that rainfall may foster conditions conducive to algal growth, especially during peak months like April and May. However, this relationship is not consistent year-round, with weaker correlations observed in some months. RS Chl-a displayed a more stable pattern, showing less sensitivity to rainfall fluctuations compared to in situ measurements.

For TUR, the RS data indicated that turbidity was highest during the OND season and lowest at the end of the MAM season, with a gradual increase during the dry season. In contrast, in situ measurements showed the highest TUR in January, followed by a trend similar to the RS data. Generally, TUR was more closely and consistently linked to rainfall than Chl-a. As rainfall increased, in situ TUR generally rose, suggesting that rainfall causes more suspended particles in the water, leading to higher turbidity. This relationship is observed throughout most of the year, with turbidity decreasing during periods of low rainfall and increasing as rainfall picks up. Similar to Chl-a, RS TUR was less variable and did not reflect rapid changes in rainfall as clearly as in situ measurements did. In comparing Chl-a and TUR, the RS turbidity concentrations performed better as they captured a similar pattern and trend between the RS and in situ monthly measurements.

## Discussion

### Water quality in Lake Victoria

The satellite RS data was first validated with in situ IMB measurements. ESA data showed a strong, statistically significant correlation with Chl-a in situ measurements, indicating high reliability for assessing chlorophyll-a concentrations. Conversely, Copernicus data showed a stronger correlation with in situ data for TUR. Both datasets originate from the OLCI sensor, but differences arise from processing options. The main distinction is that Copernicus data is aggregated over 10 days, while ESA data is updated daily.

The differences between RS and in situ datasets can be attributed to various factors, including disparities in spatial and temporal resolution, retrieval algorithms, and the transformation from TSI to Chl-a for Copernicus data. Additionally, the accuracy of satellite-derived Chl-a and TUR estimates can be compromised by cloud cover, atmospheric correction, and calibration errors (Matthews et al., 2012). Algorithms are typically tuned to in situ observations and weighted by similarity to 13 “optical water types” (Liu et al., 2021). Once established for a specific water type, they can be applied globally. However, the system is sensitive to local bias in the training data; if calibration data is from a single source, it influences algorithm tuning, causing errors when applied elsewhere. This is particularly significant for tropical lakes, where the lack of regular in situ water quality data often hinders their inclusion in algorithm calibration and validation. Moreover, atmospheric correction for inland water bodies using global satellite products is still imperfect and difficult to validate due to limited in situ radiometric reference data (Liu et al., 2021). Measuring Chl-a in turbid and eutrophic areas is also challenging due to suspended sediment, especially along shores and major hotspots like the Winam Gulf (Ambrose-Igho et al., 2021; Baltodano et al., 2022). Furthermore, in situ measurements can be affected by sensor drift, calibration and measurement errors, and environmental factors such as fouling, which impact their accuracy for validation purposes (Mills & Fones, 2012).

However, despite the above challenges, the combination of statistical metrics and visualizations provided a comprehensive understanding of the correlation between the RS and in situ data. Improving the spatiotemporal coverage of in situ measurements and optimizing retrieval algorithms for RS would improve the correlation (Garaba et al., 2014; Gidudu et al., 2018). Enhancements in the precision of RS methods, both in terms of radiometric, spatial, and spectral aspects, along with the growing abundance of satellite images captured across various locations and time periods, will also continue elevating the capabilities for extensive monitoring of optically active water quality parameters such as turbidity and chlorophyll-a (Gholizadeh et al., 2016). To fill the gap between uncertainties with global satellite products, it is anticipated that the results of this research will provide feedback to the agencies in charge of the Copernicus and ESA datasets on the accuracy of these datasets to tropical inland water quality.

The majority of the lake was classified as oligotrophic from 2005 to 2019 with eutrophic-mesotrophic conditions along the shores indicating increased nutrient loading which is likely attributable to the increment in rainfall extremes that have become more frequent in recent years (Evans et al., 2020). The most vulnerable part of the lake is the Winam Gulf in Kenya which had mesotrophic conditions from 2010 as well as very high turbidity levels in 2020 that can be credited to rapid urban development and agricultural practices such as intensive use of fertilizers and pesticides (Fusilli et al., 2014). Turbidity in the lake remained relatively stable in the inner waters over the study period, but the shores experienced significant variations and poor water quality likely caused by climate change coupled with human activities as witnessed in Lake Poyang (Feng et al., 2012). Therefore, it is crucial to quantify the impacts of land cover transformations on hydrological factors such as runoff, sediment load, and nutrient fluxes for accurate water quality assessment and management (Baltodano et al., 2022). Additionally, it is worth noting that TUR and Chl-a across the whole lake exhibited a nearly similar temporal and spatial distribution pattern suggesting that TUR may primarily be influenced by algae rather than sediment load (Ambrose-Igho et al., 2021).

Examining seasonal variations, there were no major differences between the MAM and OND wet seasons, both of which showed good inner water quality but meso-eutrophic conditions along the shores. This is attributed to runoff from agricultural and urban areas, soil erosion with nutrient-rich sediment, and fluctuations in nutrient concentrations (Rashmi et al., 2022). However, the dry season exhibited more meso-eutrophic conditions, similar to TUR observations at pollution hotspots like the Winam Gulf. This can be explained by reduced water levels and increased evaporation concentrating nutrients as well as limited outflow causing nutrient accumulation thus increasing nutrient availability (Lukhele & Msagati, 2024). These factors together with human activities such as agricultural runoff, wastewater discharge, and industrial effluents introducing additional nutrients could enhance nutrient concentrations and biological productivity (Dube et al., 2017).

### Precipitation variability across the LVB and its impacts on water quality

The analysis showed a high spatial variability of precipitation across the whole LVB for the period of study with the lowest amount received in the Southeastern part of the basin. The highest rainfall was recorded in 2020 whereas the lowest in 2000 and 2005. The LVB has reportedly been receiving an increase in rainfall over the past decades as shown in previous studies (Akurut et al., 2014; Awange et al., 2013; Kizza et al., 2009; Nkwasa et al., 2022). The Mann–Kendall (MK) test showed that the “long rains” season received less rainfall over the years with the “short rains” season receiving more especially in December. The dry season was also becoming wetter, more so in July indicating a shift in the on-set and cessations of the MAM and OND seasons (Evans et al., 2020). This shows that there are climatic changes taking place across the basin similar to the findings from the MK test done by Kizza et al. (2009), that showed positive trends dominating the Lake Victoria Basin over the twentieth century.

The comparison of the chlorophyll-a concentrations and turbidity of the Winam Gulf in Kenya to precipitation showed that the water quality parameters increased with rainfall. This rise is possibly due to runoff carrying sediments and nutrients from agricultural areas, a major land use around the gulf (Calamari et al., 2006). Consequently, nutrient flux promotes eutrophication, reflected in elevated chlorophyll-a and higher turbidity. The improvement in TUR concentrations in 2022 cannot be solely attributed to reduced rainfall necessitating other factors influencing TUR such as better long-term sustainable solid/liquid waste management practices set up by the Kenyan Government (Kundu et al., 2017). The seasonal analysis showed that for Chl-a, an increase in rainfall resulted in higher concentrations which could be explained by the increased nutrient availability and hence subsequent algae growth. An almost similar relationship was obtained with TUR. This trend is ascribed to the impact of intense precipitation events on erosion, particularly in urban and agricultural areas, as well as flooding, which agitates sediment that has been deposited on the bed of the tributary rivers or lake (Rui et al., 2012).

Looking at the seasonal variations in the IMB, Chl-a concentrations followed the same pattern except for the second wet season, where there was a sharp decline in November. The observed phenomenon is explained by the fact excessive rainfall increases nutrient runoff, promoting algae growth and consequently elevating Chl-a levels. However, the downside is that excessive algae growth leads to oxygen depletion due to the decomposition of organic matter, limiting further growth and resulting in a reduction in Chl-a concentration (Varadharajan & Soundarapandian, 2014). Furthermore, excessive rainfall and runoff also reduce light penetration due to heightened turbidity as well as altering water temperature and salinity, further impacting phytoplankton growth, and resulting in decreased chlorophyll-a levels (Acharyya et al., 2020; Nair & Nayak, 2023). We also note that built-up areas dominate the IMB as seen in Fig. 2, likely having a modest impact on Chl-a concentration. The seasonal analysis with precipitation data in the IMB indicated that turbidity levels were lowest during periods of abundant precipitation and highest with moderate rainfall. This could be explained by the fact that during heavy rainfall events, the rapid flux of water can effectively dilute the concentration of suspended particles, causing a temporary spike in turbidity (Lawler et al., 2006). In addition, during moderate rainfall events, slower water flow increases sediment accumulation, leading to a sustained rise in turbidity (Li et al., 2024). Additionally, the IMB’s location at the River Nile inlet from L. Victoria, which drains rainwater from major cities, affects flow flux, kinetic energy, and sediment transport, further influencing turbidity (Li et al., 2024). This phenomenon indeed goes to prove that the extent of rainfall’s influence on water quality is affected by various factors, such as rainfall intensity and duration, land use practices, and lake characteristics (Zhang et al., 2019), which explains the differences in the dynamics of water quality in the IMB and the Winam Gulf.

We observed that TUR trends from in situ and Copernicus RS datasets were more similar than those for Chl-a. This is likely due to the challenges in accurately retrieving Chl-a via satellites, especially in eutrophic waters (Ambrose-Igho et al., 2021). Despite notable differences between in situ and RS datasets, the Copernicus dataset successfully captured TUR trends. Chl-a trends were also fairly well represented by the ESA dataset. This analysis supports the reliability of RS data as a complement to ground-based data, particularly in data-scarce regions, and underscores the potential of integrating RS and in situ data to enhance environmental monitoring and management.

### LULC changes in the LVB and their influence on water quality in the lake

The basin’s land cover changes were mostly conservative during 2000–2020, with minimal losses and gains observed except for built-up areas and bare land as in the various land cover maps at a 5-year interval in the Supplementary Material (Fig. S9). The land cover changes align with prior research, with slight variations in the land classification and spatial and temporal scales (Berakhi et al., 2015; Kiggundu et al., 2018; Mugo et al., 2020; Onyango et al., 2021). The increase in built-up areas in the Lake Victoria Basin is attributed to population growth, urbanization, and government policies promoting infrastructure development (Onyango & Opiyo, 2022). Conversely, the loss of bare land can be explained by the increase in constructed areas. Similar drivers have been demonstrated to influence extensive LULC changes in various regions of the LVB (Ebanyat et al., 2010; Wasige et al., 2013). The increase in forested areas is accredited to afforestation drivers in the region since the 1980s (Mugure & Oino, 2013).

For the LULC change analysis in the Winam Gulf, there was a slight increment in agricultural areas from 2005 till 2010. An increase in agricultural fields leads to increased runoff of nutrients like nitrogen and phosphorus which result in algae blooms that cause loss of biodiversity, alterations in food chain dynamics, and deterioration of fisheries (Verschuren et al., 2002; Wasige et al., 2013). The decline in Chl-a concentrations after 2010 can be attributed to the reduction in agricultural areas near the gulf. In contrast, the rise in turbidity between 2016 and 2020 can be attributed to increased built-up areas, which intensify stormwater runoff and flooding events.

The comparison of water quality parameters with LULC changes in the IMB revealed that the increase in TUR levels, particularly in 2018, could be explained by the increase in built-up areas at the expense of wetlands and forests over the past two decades, leading to increased sediment transport. Bhaskar and Gidudu (2020) similarly reported a 119% rise in impervious surface area and a 12% decline in vegetative cover from 1995 to 2019, which was associated with higher Chl-a concentrations in the bay. However, the elevated Chl-a levels in the IMB, especially in the recent decade, could not be fully explained by land use changes, as agricultural areas have decreased. Murchison Bay, the primary source for drinking water supply for Kampala city, also receives surface runoff, municipal and industrial waste, and sewage effluents. Over time, the bay has been affected by wetland degradation, amplified pollution from Kampala, water hyacinth proliferation, and reduced water levels (Ssebiyonga et al., 2013). Hence, there is a need to investigate the possible impacts of these influences on the quality of water.

The analysis of NDVI data provided valuable insights into seasonal vegetation coverage changes for the Inner Murchison Bay and the Winam Gulf. The increased vegetation coverage between 2016 and 2021 around the Winam Gulf showed the positive impact of favorable climatic conditions on vegetation health and density. For the IMB, vegetation density was generally greater during the MAM season compared to the OND season in both years which also proved the impact of more rainfall on vegetation density as the MAM season received more rainfall than the OND.

### Limitations

The results of this research, while aligning with previous studies, are hampered by several limitations. Firstly, the limited time span of available satellite and in situ data restricted our ability to analyze long-term trends. The comparison between global satellite products and in situ data revealed moderate correlations, ranging from 0.41 to 0.73. Consequently, we had to selectively work with the datasets that exhibited better correlations for each parameter, which further constrained the time ranges we could study for each dataset. This selective approach, while necessary, introduced limitations in our analysis of temporal trends. Secondly, only the influence of precipitation and LULC changes on water quality in the Winam Gulf and IMB was examined. While the increased built-up areas in the Winam Gulf and the prominent agricultural practices may contribute to TUR and Chl-a concentrations, substantial rainfall was a better explanatory factor for the water quality patterns and trends. This showed that rainfall plays a crucial role in influencing water quality dynamics, particularly in areas with unrestricted human activities like urbanization and agriculture (Tuladhar et al., 2019). In the inner Murchison Bay, high Chl-a levels could not be solely explained by land use changes, suggesting additional pollution sources such as industries and sewage effluents to be responsible for the elevated Chl-a and TUR levels. Furthermore, examining water quality from the tributary rivers of the lake is essential to comprehensively understand the influence of land cover changes on water quality, as rivers serve as conduits for transporting nutrients and pollutants from land to the lake.

In addition, mixing processes in the lake could be a factor to consider as a study by Hecky (1993) showed that some of the causes of degradation in Lake Victoria are shallow mixing depths because of climate change and low flushing times. Okungu et al. (2005) suggested that winds from a southerly direction dominated mixing and nutrient transport processes at Winam Gulf, whereas winds from the northeast were less important in the annual transport of nutrients and sediments. A mixing box model by Gikuma-Njuru et al. (2013) also revealed that most nutrients from river inflows and municipal sources entering the Winam Gulf were retained within the gulf, with only a small portion transferred to the main lake. For the IMB, wind-induced mixing influenced its water quality (Akurut et al., 2017; Ssebiyonga et al., 2013). Thus, it is vital to explore the wind dynamics over the lake as well as the flushing and exchange patterns between the bays, gulfs, and offshore waters.

There are quite a variety of other natural and human-induced factors that influence the Chl-a and TUR concentrations of a water body such as an excessive nutrient load from point and diffuse sources, raw or semi-treated effluents from industries and wastewater treatment facilities, and physical factors such as the lake’s flushing rate, loss of biodiversity, and climate change. According to Kaijanangoma (2020), effluents discharged into Lake Victoria are primarily raw or partially treated. Treatment ponds installed at most wastewater treatment facilities and industries are inadequate in size with a short retention time. As a result, raw and semi-treated wastewater is released into the lake, leading to contamination and harm to fish breeding sites due to high BOD. While nutrient load is a key driver of eutrophication, factors like temperature, precipitation, wind velocity, and solar radiation can increase the risk. Climate change, characterized by shifts in precipitation, temperature, wind speed, and solar radiation, directly impacts water quality by altering stream flow and water temperatures (Rolighed et al., 2016; Rui et al., 2012). Studies conducted by Cooke et al. (2016) and Krzyk et al. (2015) revealed that certain lakes are exposed to tidal effects and contamination because of their outlets. These lakes undergo frequent renewal, making them less susceptible to eutrophication. Consequently, the polluted water in such lakes is efficiently purified due to its short residence time. Thus, while this analysis examines land use and precipitation, it is important to acknowledge that other environmental factors specific to the lake’s location can also influence water quality (W. Liu et al., 2010).

## Conclusion

With a focus on two hotspots for water pollution, Winam Gulf and inner Murchison Bay, this study was able to use global satellite products to examine the relationship between changes in land use and rainfall in the Lake Victoria basin to explain variations in the lake’s water quality. A comparison between satellite and in situ data revealed that ESA satellite data was more reliable for measuring Chl-a, while Copernicus data was better for TUR. Seasonal analysis in Winam Gulf showed that Chl-a and TUR generally increased with rainfall. This is due to increased runoff and erosion during heavy rains, which transport pollutants and nutrients from agricultural lands into the lake. In urban areas like IMB, characterized by extensive impervious surfaces, turbidity increased during moderate rainfall (OND, wet season) but decreased during heavy rainfall (MAM, wet season) possibly due to sediment dilution.

The study also highlighted significant land use changes in both hotspots, with increased built-up areas replacing bare land in Winam Gulf and wetlands and forests in IMB. These built-up areas contribute to higher nutrient and pollutant runoff during heavy rains, leading to increased TUR and Chl-a concentrations. The analysis revealed that while land use changes had a positive correlation with Chl-a and TUR concentrations in the Winam Gulf and IMB, changes in rainfall provided a better explanation for the observed patterns. However, in the IMB, high Chl-a values could not be attributed solely to changes in precipitation and land use, indicating the influence of additional factors such as poor wastewater treatment and industrial discharges. This highlights the need to recognize the complex interactions between land use, urbanization, and climate change in affecting water quality.

Effective water management strategies may need to address multiple factors simultaneously. With the availability of remote sensing data, it is crucial to explore other factors like wind that influence water quality in lakes and rivers. Future research could investigate the impact of wastewater treatment effluents and wind speed on chlorophyll-a and turbidity, respectively. Improving the algorithms for retrieving water quality parameters from RS data would also enhance the accuracy of results and their alignment with ground measurements, increasing reliability and validity. In addition, there is also a need to improve the resolution of the global satellite products and the availability of more in situ water quality data to use in the validation of the RS data especially for tropical lakes.

## Supplementary Information

Below is the link to the electronic supplementary material.

Supplementary file1 (PDF 1341 KB)

## Data Availability

All datasets used in this study are openly available and are accessible from these links: “VITO” RS data for turbidity and trophic state index is available from https://land.copernicus.eu/global/products/lwq. “ESA” RS data for turbidity and chlorophyll-a is available from https://climate.esa.int/en/projects/lakes/data/ . CHIRPS rainfall data is available from https://www.chc.ucsb.edu/data/chirps. ESA-CCI Land cover maps are available from https://www.esa-landcover-cci.org. Landsat 8 images are available from https://earthexplorer.usgs.gov/. Analysis and processing of data was done using Python scripts. Scripts can be obtained from the corresponding author upon request. “Copernicus” RS data for turbidity and trophic state index is available from https://land.copernicus.eu/global/products/lwq. “ESA” RS data for turbidity and chlorophyll-a is available from https://climate.esa.int/en/projects/lakes/data/. CHIRPS rainfall data is available from https://www.chc.ucsb.edu/data/chirps. ESA-CCI Land cover maps are available from https://www.esa-landcover-cci.org. Landsat 8 images are available from https://earthexplorer.usgs.gov/. Analysis and processing of data were done using Python scripts. Scripts can be obtained from the corresponding author upon request.
