# Supplementary material for: Linking land use and precipitation changes to water quality changes in Lake Victoria using earth observation data
Source: Environ Monit Assess. 2024 Oct 25;196(11):1104. doi: 10.1007/s10661-024-13261-2 (PMC11511718; doi:10.1007/s10661-024-13261-2)
Supplement: Supplementary file 1 — Supplementary file1 (PDF 1341 KB) [file 10661_2024_13261_MOESM1_ESM.pdf]

# Linking Land Use and Precipitation Changes to Water Quality changes in Lake Victoria Using Remote Sensing

Maria Theresa Nakkazi[1], Albert Nkwasa[2,1], Analy Baltodano Martinez[1] and Ann van Griensven[1,3]

[1]Department of Water and Climate, Vrije Universiteit Brussel (VUB), 1050, Brussels, Belgium
[2]Water Security Research Group, Biodiversity and Natural Resources Program, International Institute for Applied Systems Analysis (IIASA), Schlossplatz 1, A-2361, Laxenburg, Austria
[3]Water Science & Engineering Department, IHE Delft Institute for Water Education, 2611 AX, Delft, The Netherlands
Correspondence: Maria Theresa Nakkazi (maria.theresa.nakkazi@vub.be)

## Supplementary Materials

**Fig. S1**: The 26 water sampling locations in the IMB and their location in reference to the lake

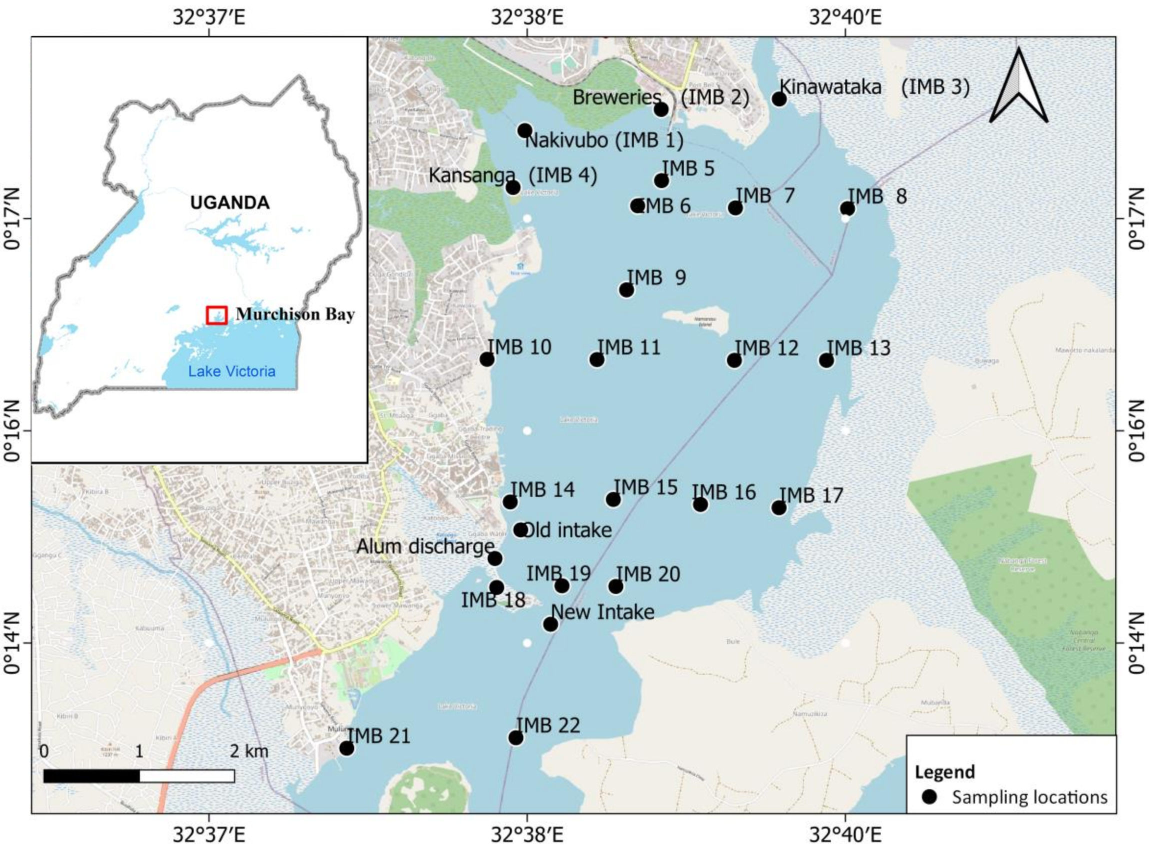

**Fig. S2**: Histograms and Boxplots showing distribution of chlorophyll-a and turbidity concentrations for In-situ, Copernicus, and ESA data in the Inner Murchison Bay for year 2018.

## Chlorophyll-a

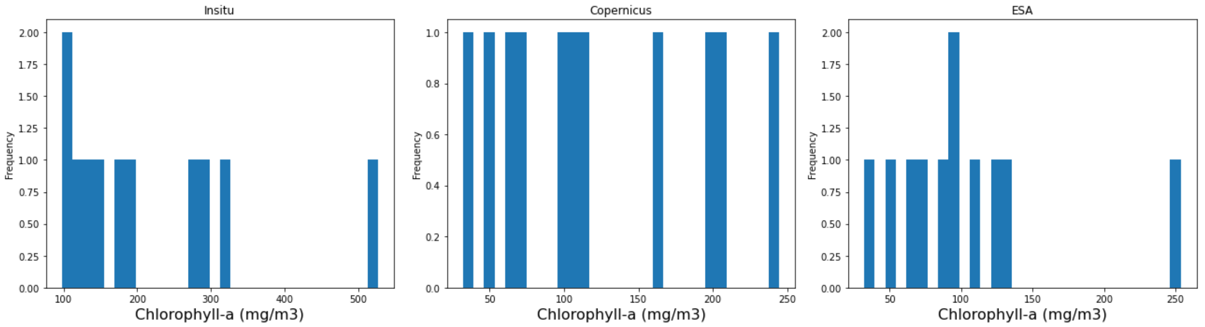


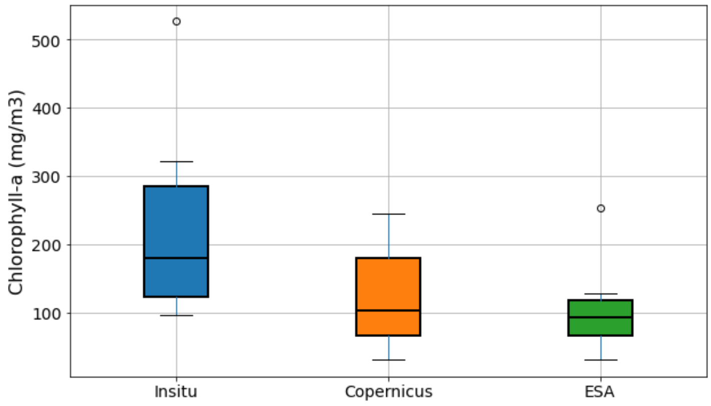


## Turbidity

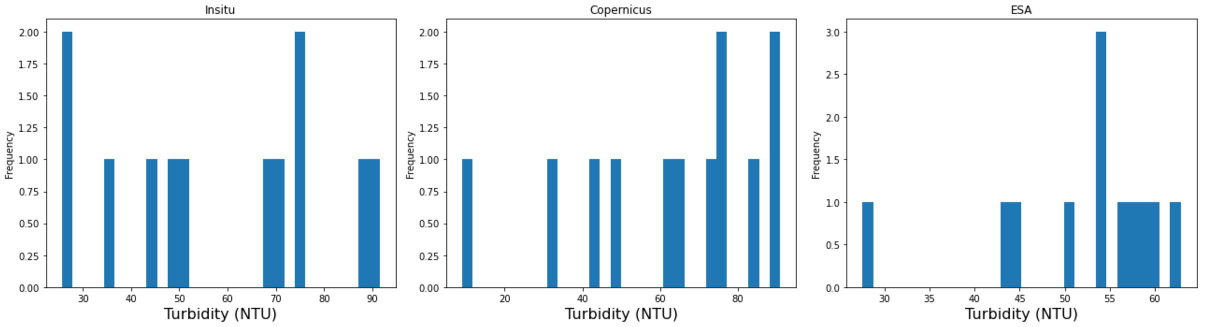


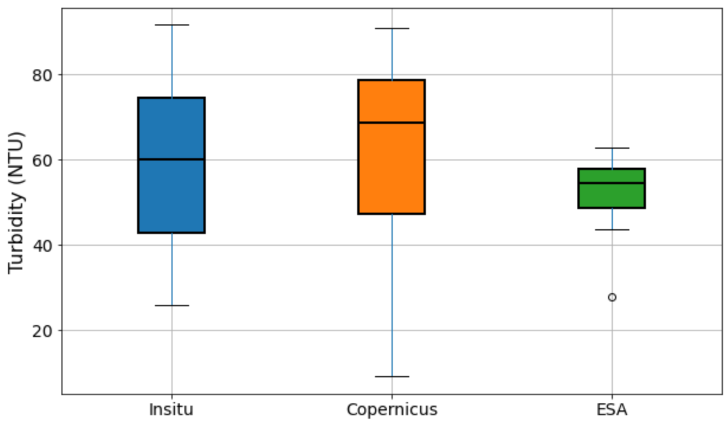

**Fig. S3**: Time series chart for chlorophyll-a and turbidity for in-situ, Copernicus, and ESA data

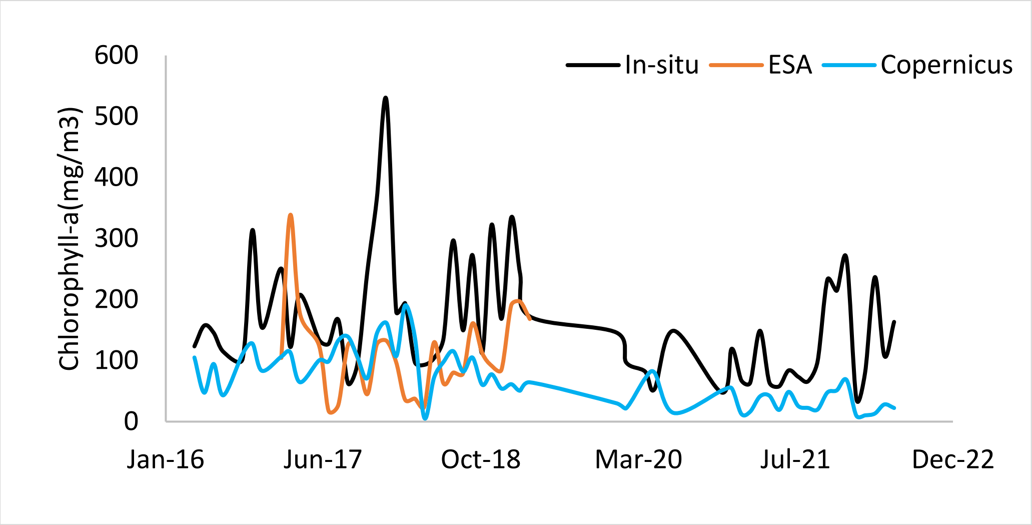


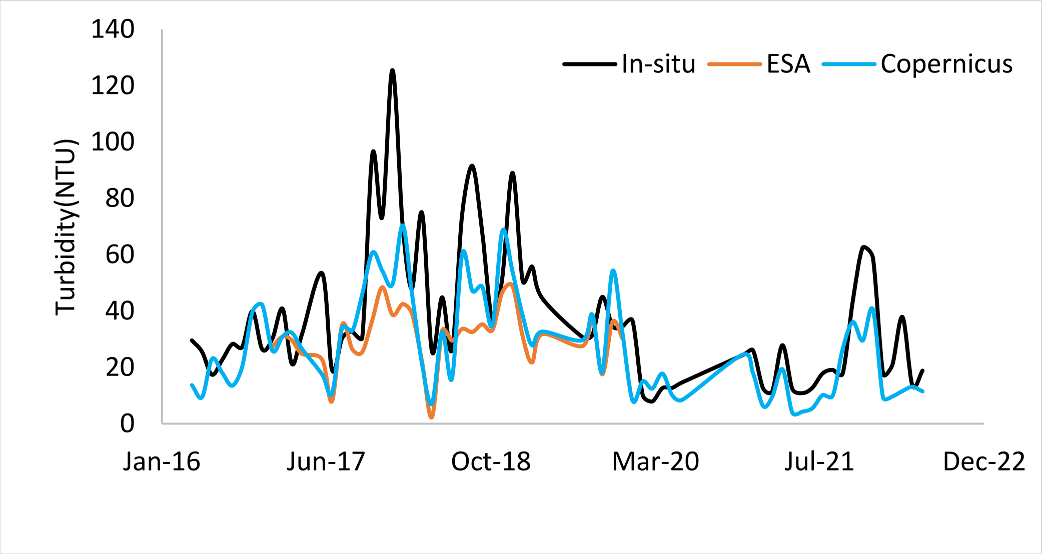


**Fig. S4:** Mean annual precipitation across the Lake Victoria Basin for the period 2000 - 2022

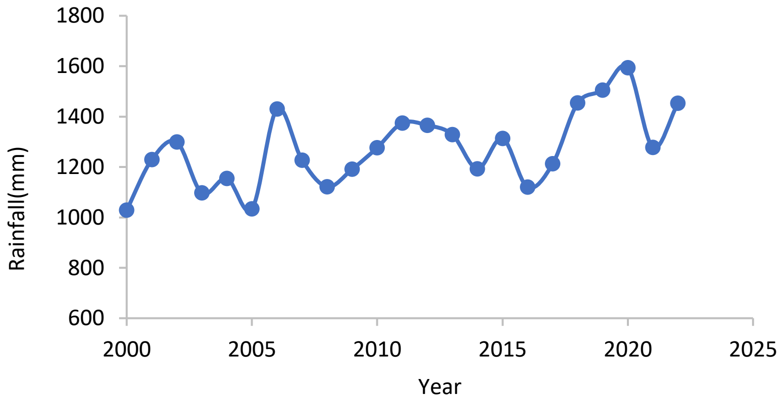

**Fig. S5:** Seasonal precipitation trends over the LVB

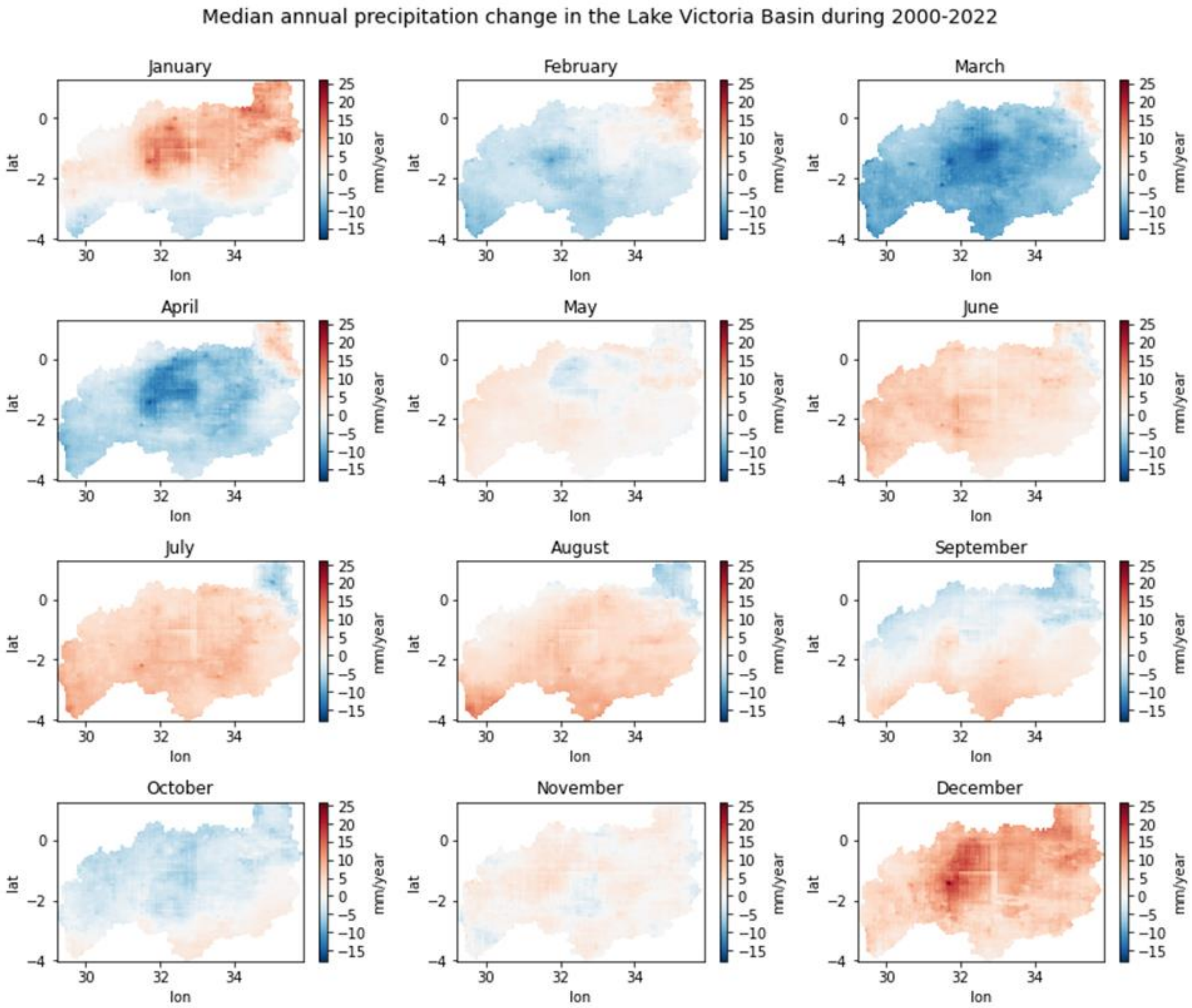

**Fig. S6:** Spatial distribution of the Sen's slope of rainfall at monthly scale

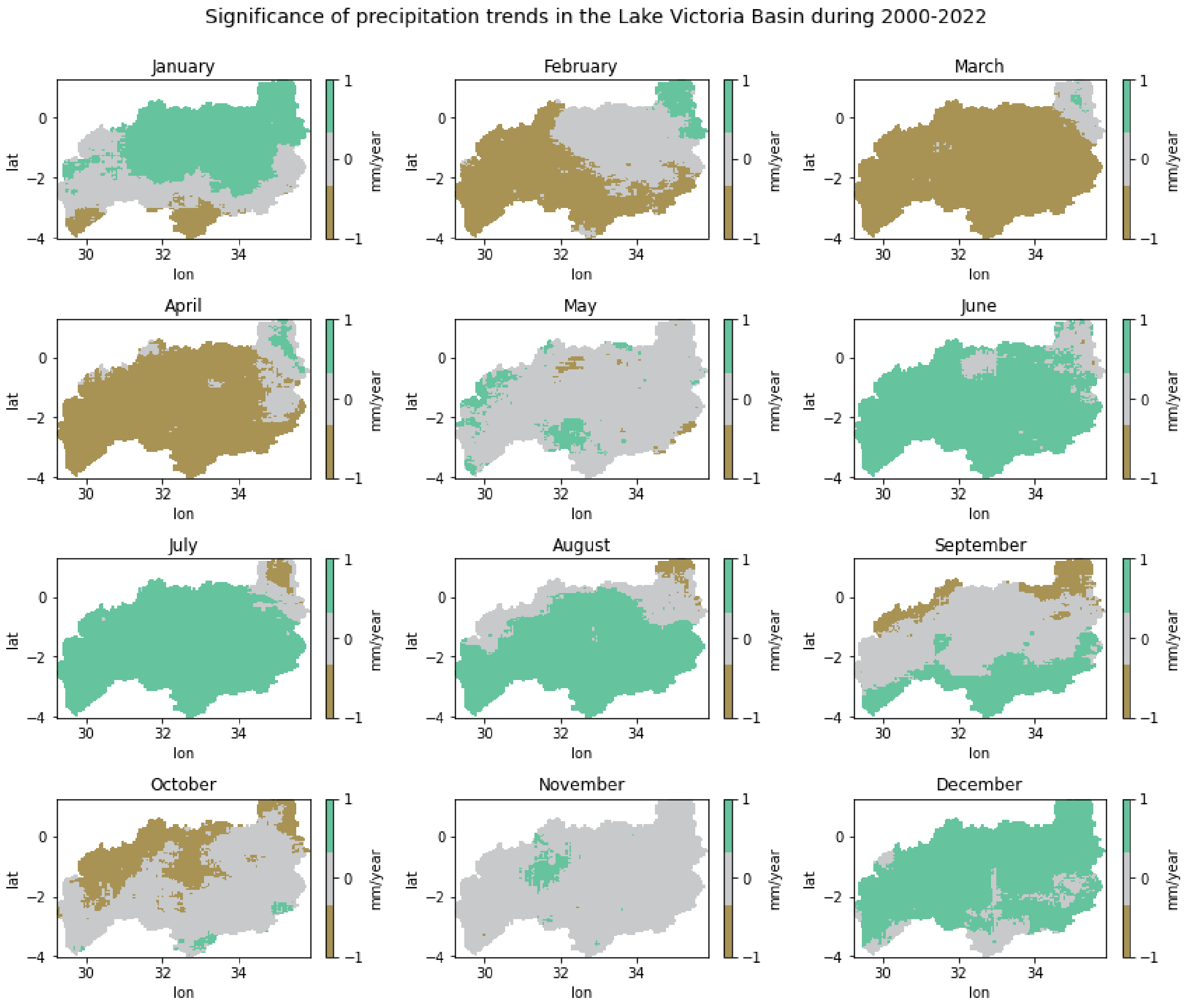


**Fig. S7:** Gain, persistence, and loss by category in percentage between 2000 and 2020

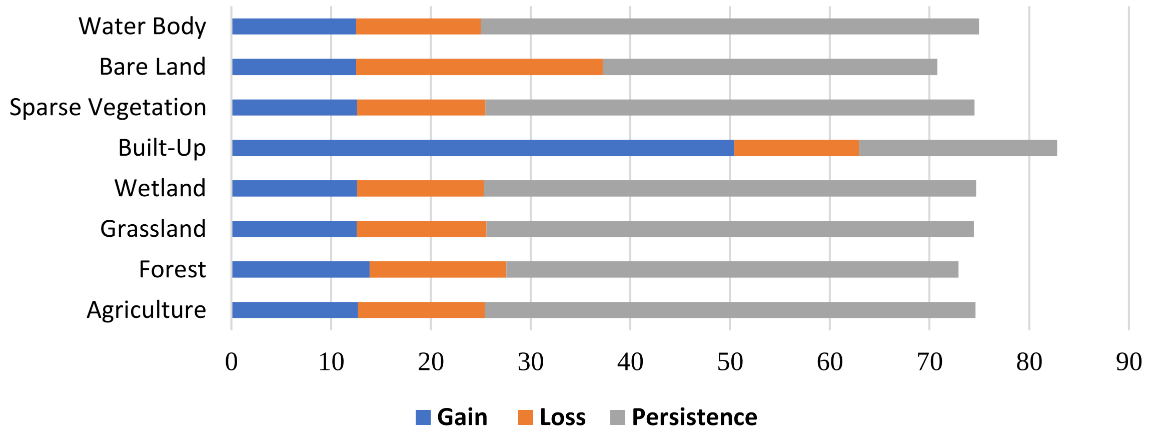

**Fig. S8:** Intensity of transitions between the different land cover types between 2000 and 2020

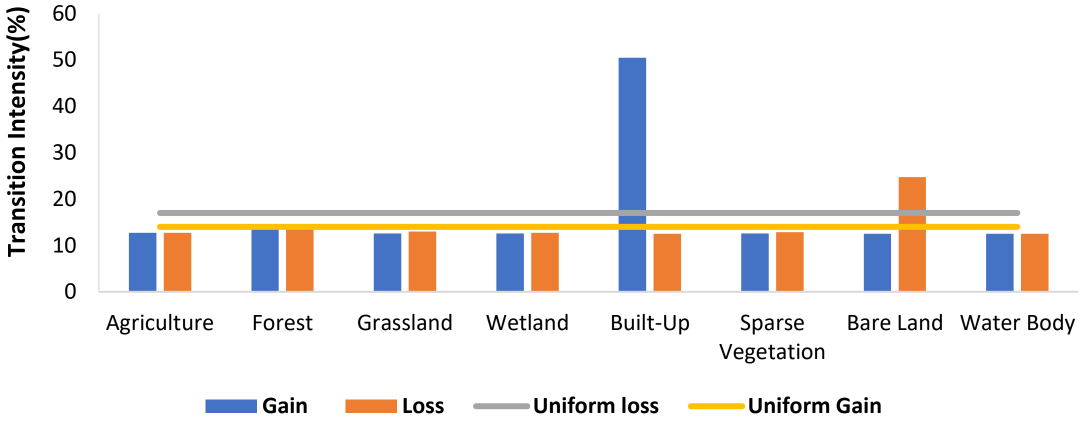


**Fig. S9:** Land cover maps of the Lake Victoria basin from 2000 to 2020 at a 5-year time step

**2000**

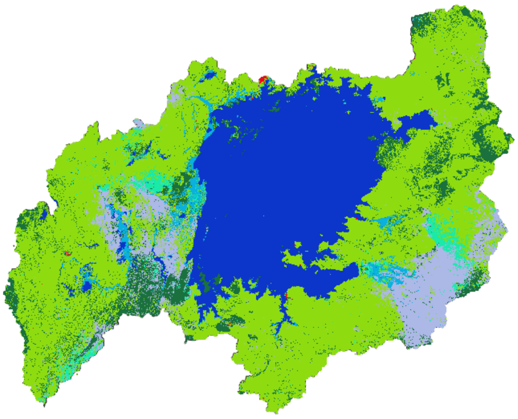

**2005**

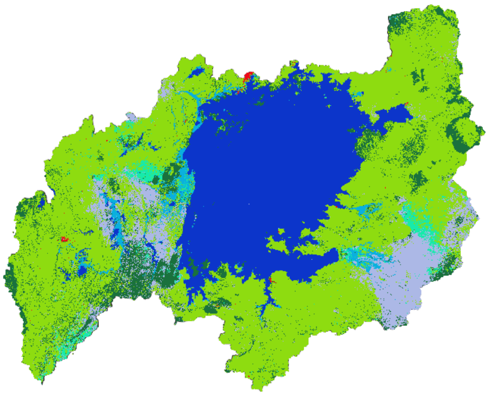

**2010**

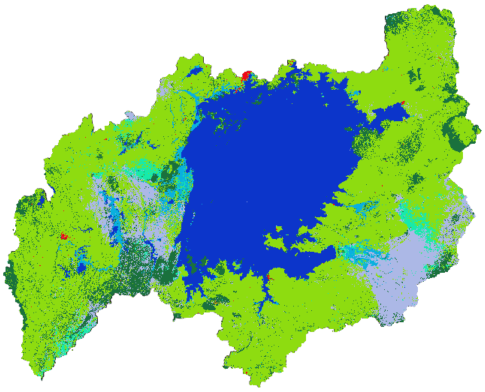

**2015**

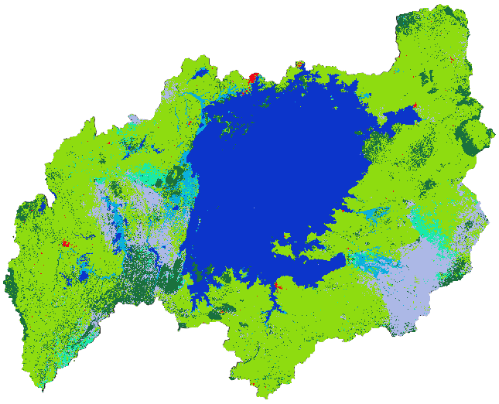

**2020**

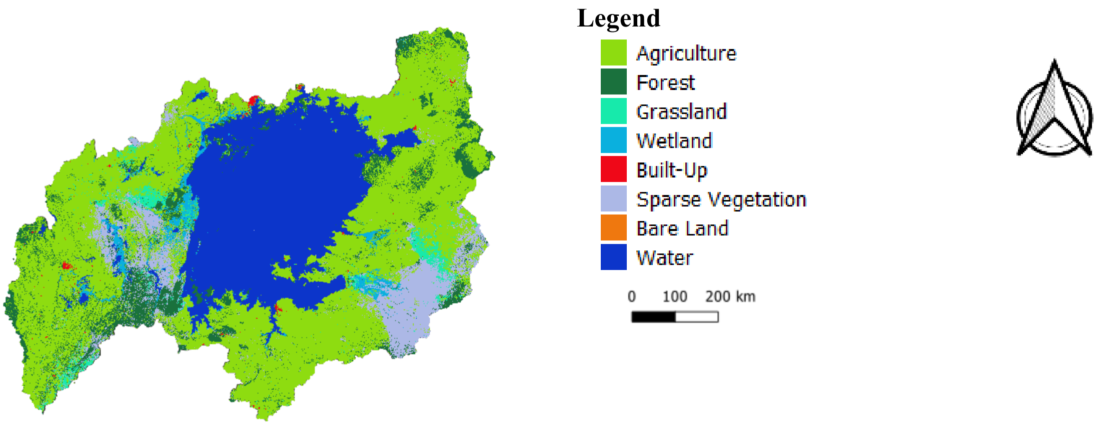


**Fig. S10:** NDVI maps of the Winam Gulf for the years 2016 and 2021 for different seasons

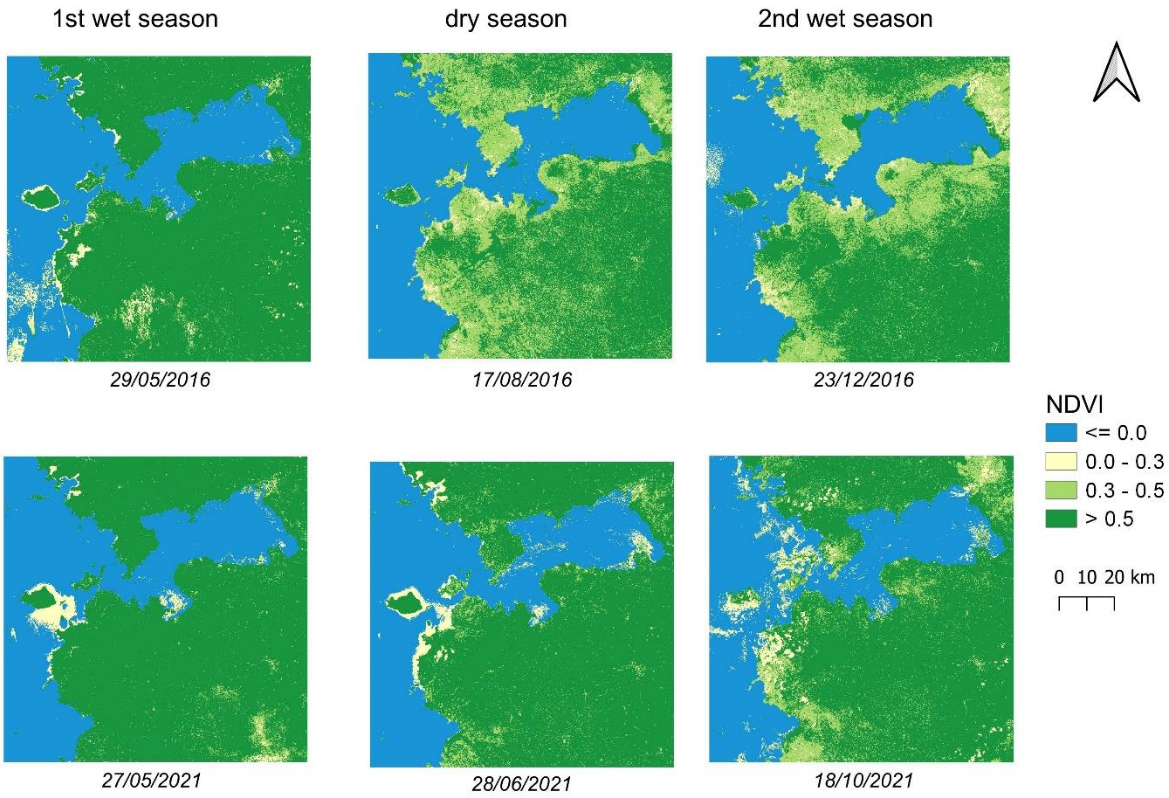

**Fig. S11:** NDVI maps of the Inner Murchison Bay (IMB) for the years 2016 and 2021 for different seasons

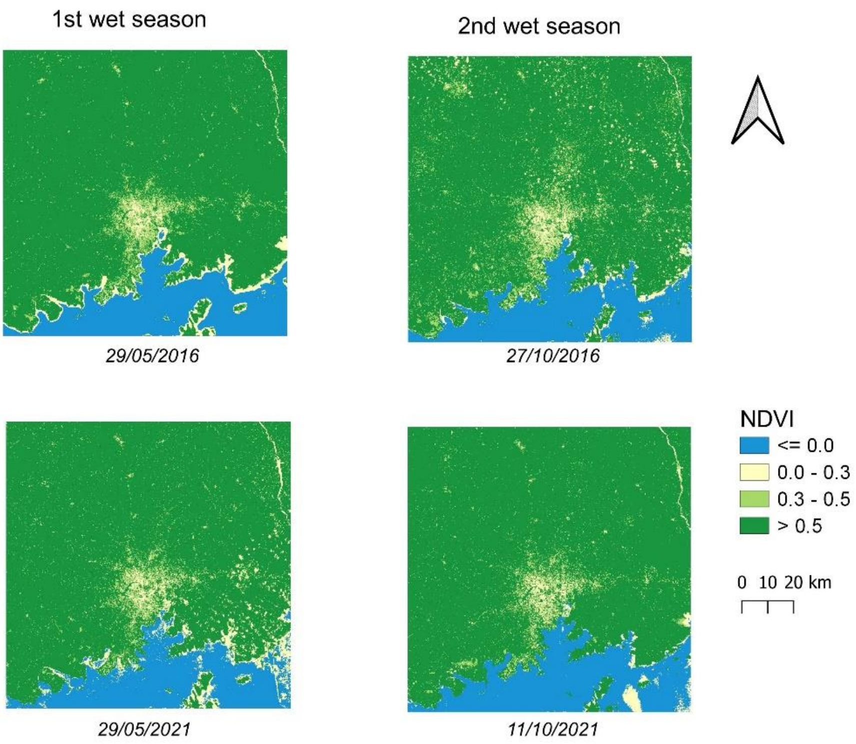


**Table S1:** List of Mathematical formulas for the statistical metrics used throughout the study

| **No.** | **Statistical Metric** | **Description** |
|---|---|---|
| 1 | Mean (Arithmetic Average) | The mean is the sum of all observations divided by the number of observations. $Mean(\mu) = \frac{1}{N}\sum_{i=1}^{N} x_i$ |
| 2 | Median | The median is the middle value of an ordered dataset. |
| 3 | Standard Deviation | The standard deviation measures the amount of variation or dispersion in a set of values. $Standard\ Deviation\ (\sigma) = \sqrt{\frac{1}{N}\sum_{i=1}^{N}(x_i - \mu)^2}$ |
| 4 | Pearson Correlation Coefficient | The Pearson correlation coefficient (r) measures the linear relationship between two variables. $r = \frac{\sum_{i=1}^{N}(x_i - \bar{x})(y_i - \bar{y})}{\sqrt{\sum_{i=1}^{n}(x_i - \bar{x})^2 \sum_{i=1}^{n}(y_i - \bar{y})^2}}$ |
| 5 | Coefficient of Variation (CV) | The coefficient of variation (CV) is a standardized measure of the dispersion of a frequency distribution. It is calculated as the ratio of the standard deviation to the mean. $Coefficient\ of\ Variation\ (CV) = \frac{\sigma}{\mu}$ |

**Table S2:** Derivation table of chlorophyll-a from TSI (adapted from Simis (2020))

| Trophic classification | Trophic State Index | Chlorophyll-a |
|---|---|---|
| Oligotrophic | 0 | 0.04 |
| | 10 | 0.12 |
| | 20 | 0.34 |
| | 30 | 0.94 |
| Mesotrophic | 40 | 2.6 |
| | 50 | 6.4 |
| Eutrophic | 60 | 20 |
| | 70 | 56 |
| Hypereutrophic | 80 | 154 |
| | 90 | 427 |
| | 100 | 1183 |

**Table S3:** Table for reclassification of land cover types from Mousivand & Arsanjani (2019)

*(Originally adapted from Land Cover CCI: product user guide, Version 2, available at https://www.esa-landcover-cci.org/?q=webfm_send/84).*

| No. | Classes considered in the study | Nomenclature used in the CCI-LC maps | |
|---|---|---|---|
| 1. | Agriculture | 10,11,12 | Rainfed Cropland |
| | | 20 | Irrigated Cropland |
| | | 30 | Mosaic cropland (>50%)/natural vegetation (tree, shrub, herbaceous cover) (<50%) |
| | | 40 | Mosaic natural vegetation (tree, shrub, herbaceous cover) (>50%)/cropland (<50%) |
| 2. | Forest | 50 | Tree cover, broadleaved, evergreen, closed to open (>15%) |
| | | 60,61,62 | Tree cover, broadleaved, deciduous, closed to open (>15%) |
| | | 70,71,72 | Tree cover, needle leaved, evergreen, closed to open (>15%) |
| | | 80,81,82 | Tree cover, needle leaved, deciduous, closed to open (>15%) |
| | | 90 | Tree cover, mixed leaf type (broadleaved and needle leaved) |
| | | 100 | Mosaic tree and shrub (>50%)/herbaceous cover (<50%) |
| | | 160 | Tree cover, flooded, fresh or brakish water |
| | | 170 | Tree cover, flooded, saline water |
| 3. | Grassland | 110 | Mosaic herbaceous cover (>50%)/tree and shrub (<50%) |
| | | 130 | Grassland |
| 4. | Wetland | 180 | Shrub or herbaceous cover, flooded, fresh-saline or brakish water |
| 5. | Settlement | 190 | Urban |
| 6. | Sparse Vegetation | 120,121,122 | Shrubland |
| | | 140 | Lichens and mosses |
| | | 150,151,152,153 | Sparse vegetation (tree, shrub, herbaceous cover) |
| 7. | Bare area | 200,201,202 | Bare areas |
| 8. | Water | 210 | Water |

**Table S4:** Transition matrix for the period between 2000 – 2010 (in bold), 2010 – 2020 (in italic) and 2000 – 2020 (underlined)

| **Land Cover Classes – 2000, 2010** | **Land Cover Classes – 2010, 2020** | | | | | | | | | |
|---|---|---|---|---|---|---|---|---|---|---|
| | Agriculture | Forest | Grassland | Wetland | Built-Up | Sparse Vegetation | Bare Land | Water Body | Gross Loss | %Uniform Loss |
| Agriculture | **127,954**<br>*128,587*<br>126,729 | **725**<br>*973*<br>1,668 | **0**<br>*2*<br>3 | **1**<br>*10*<br>3 | **151**<br>*34*<br>382 | **26**<br>*12*<br>71 | **-**<br>-<br>- | **23**<br>*2*<br>26 | **128,880**<br>*23,268*<br>128,880 | **13**<br>*13*<br>13 |
| Forest | **1,716**<br>*148*<br>1,841 | **22,166**<br>*23,062*<br>21,987 | **10**<br>*2*<br>12 | **30**<br>*10*<br>37 | **69**<br>*34*<br>104 | **112**<br>*12*<br>121 | **-**<br>-<br>- | **5**<br>*2*<br>7 | **24,109**<br>*23,268*<br>24,109 | **14**<br>*13*<br>14 |
| Grassland | **83**<br>-<br>82 | **54**<br>*60*<br>115 | **5,477**<br>*5,431*<br>5,406 | **-**<br>-<br>- | **6**<br>*9*<br>15 | **-**<br>*1*<br>1 | **-**<br>-<br>- | **1**<br>*2*<br>3 | **5,622**<br>*5,503*<br>5,622 | **13**<br>*13*<br>13 |
| Wetland | **-**<br>-<br>- | **52**<br>*49*<br>101 | **-**<br>-<br>- | **7,150**<br>*7,141*<br>7,097 | **1**<br>*1*<br>2 | **-**<br>-<br>- | **-**<br>-<br>- | **16**<br>*4*<br>20 | **7,220**<br>*7,196*<br>7,220 | **13**<br>*13*<br>13 |
| Built-Up | **-**<br>-<br>- | **-**<br>-<br>- | **-**<br>-<br>- | **-**<br>-<br>- | **175**<br>*417*<br>175 | **-**<br>-<br>- | **-**<br>-<br>- | **-**<br>-<br>- | **175**<br>*417*<br>175 | **13**<br>*13*<br>13 |
| Sparse Vegetation | **83**<br>*1*<br>80 | **266**<br>*253*<br>521 | **7**<br>*1*<br>*8* | **-**<br>-<br>- | **13**<br>*10*<br>23 | **21,470**<br>*21,345*<br>21,401 | **-**<br>-<br>4 | **-**<br>-<br>68,787 | **21,839**<br>*21,610*<br>21,836 | **13**<br>*13*<br>13 |
| Bare Land | **-**<br>-<br>- | **-**<br>-<br>- | **-**<br>-<br>- | **-**<br>-<br>- | **0**<br>*4*<br>4 | **-**<br>-<br>- | **9**<br>*4*<br>4 | **-**<br>-<br>- | **9**<br>*9*<br>9 | **13**<br>*24*<br>25 |
| Water Body | **4**<br>-<br>4 | **5**<br>-<br>- | **7**<br>*1*<br>8 | **15**<br>*0*<br>15 | **1**<br>*1*<br>2 | **2**<br>-<br>2 | **-**<br>-<br>- | **68,733**<br>*68,776*<br>68,731 | **68,767**<br>*68,778*<br>68,767 | **13**<br>*13*<br>13 |
| Gross Gain | **129,840**<br>*128,736*<br>128,736 | **23,268**<br>*24,397*<br>24,397 | **5,503**<br>*5,437*<br>5,437 | **7,196**<br>*7,151*<br>7,151 | **417**<br>*708*<br>708 | **21,670**<br>*21,401*<br>21,401 | **9**<br>*4*<br>4 | **68,778**<br>*68,787*<br>68,787 | **256,621**<br>*256,621*<br>256,621 | |
| % Uniform Gain | **13**<br>*13*<br>13 | **13**<br>*13*<br>14 | **13**<br>*13*<br>13 | **13**<br>*13*<br>13 | **30**<br>*21*<br>50 | **13**<br>*13*<br>13 | **13**<br>*13*<br>13 | **13**<br>*13*<br>13 | | |
